# Search for Low-Periodic Substructures in Crystalline Solids: A Novel Approach

**DOI:** 10.1021/acsami.6c03558

**Published:** 2026-05-05

**Authors:** Pavel N. Zolotarev, Davide M. Proserpio, Davide Campi

**Affiliations:** † Dipartimento di Scienza dei Materiali, 9304 Università degli Studi di Milano-Bicocca Via R. Cozzi 55, 20125 Milano, Italy; ‡ Dipartimento di Chimica, Università degli Studi di Milano, Via Golgi 19, 20133 Milano, Italy

**Keywords:** two-dimensional materials, exfoliation, high-throughput screening algorithm, bond valence method, data mining, machine learning, density functional theory

## Abstract

The discovery of new 2D materials is vital for advancing electronics and quantum technologies. As most 2D materials originate from layered bulk structures, identifying exfoliable crystals and estimating the energy required to isolate a single layer are critical steps. To address this issue, we developed a robust and computationally cheap approach based on the crystal graph construction via Voronoi partition, interaction strength estimation via bond valence theory, and the iterative removal of weak links while tracing the periodicity changes. We validated our method against literature and ab initio results proving that it can reliably identify layers and provide an approximate estimate of the interlayer binding energy suitable as a screening parameter. We subsequently applied it to analyze a large set of 48,504 preselected experimental crystal structures, uncovering 694 previously unreported 2D materials belonging to 530 different structural prototypes. Finally, we used ab initio simulations to offer an overview the structural and electronic properties of the isolated layers.

## Graphical abstract

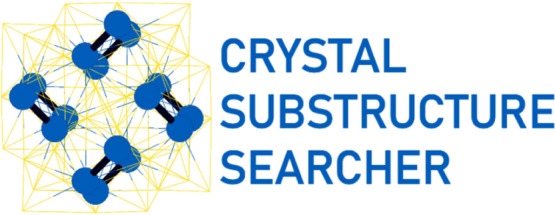

## Introduction

Low-dimensional (1D and 2D) or better termed low-periodic (1-periodic and 2-periodic, respectively) materials represent some of the most promising candidates for beyond-silicon electronic, optoelectronic, and quantum computing applications. In recent years, their widely recognized importance has catalyzed an intense effort within the scientific community to discover and characterize novel low-periodic materials with potentially groundbreaking properties. Among various discovery strategies, one particularly fruitful approach involves the systematic analysis of 3-periodic (bulk) crystal structures with the specific aim of identifying embedded low-periodic substructures that could be isolated and further exploited.^1–4^

When considering well-known layered materials such as graphite or transition metal chalcogenides, the corresponding layered substructures can be easily identified, even by the straightforward visual analysis of the bulk structures. However, systematic discovery on a larger scale demands more rigorous and automated methods. In response to this challenge, researchers have developed numerous automatic substructure identification methods. These methodologies broadly fall into several categories based on their underlying principles. The first category encompasses methods relying on the calculation of physical properties of bulk crystals that indicate the existence of low-periodic substructures. These include: (i) analysis of force constants^5^ reflecting the strength of interatomic interactions. The periodicity of the substructures obtained at the selected force constant threshold value determines the periodicity of the crystal (ii) Young’s modulus analysis,^6^ which identifies anisotropies in elastic properties that often reflect the periodicity of the substructures. A second category involves techniques, such as (iii) the straightforward comparison of experimental and theoretically optimized structures,^7^ which can reveal weak interactions between substructures through peculiarities of structure relaxation patterns. While these approaches have demonstrated significant success in identifying low-periodic substructures, they have some practical limitation. In particular, they heavily rely on density functional theory (DFT) calculations, thus demanding extensive computational resources that limit their broad application to large structural databases.

A third important and widely used category comprises simple interatomic distance-based algorithms, with several distinct implementations.^1,8,9^ The fundamental concept underlying these algorithms is rather straightforward: the algorithm searches for networks of strong bonds within the crystal structure and calculates the periodicity of the motifs identified. If the identified network of strong bonds exhibits periodicity lower than 3-p, then the crystal can be classified as containing a corresponding layer (2-p) or chain (1-p) substructure. In practice, interatomic distance-based algorithms employ van der Waals (*R*_vdW_) or covalent (*R*_cov_) radii systems to only **qualitatively estimate bond strength**. Within this framework, a bond either exists (if the interatomic distance *r*_*ij*_ is less than the sum of relevant radii *R* plus a small tolerance Δ) or does not exist (if *r*_*ij*_ exceeds that sum), thereby allowing the construction of the network of strong bonds. However, these methods have some drawbacks, including the need for appropriate selection of reference radii system and adjustable Δ parameters that can significantly influence the results. Moreover, such methods do not provide a direct estimate of the energy required to isolate the identified layers and thus often need to be complemented by DFT calculations. As a result, there is a clear lack of a low-cost, widely applicable method capable of providing quantitative estimates of exfoliability.

In the field of crystal chemistry, bond valence (BV) theory has long served as a valuable and well-established method for crystal structure analysis.^10^ Nowadays, BV theory applications include validation of structure solutions, estimation of oxidation states, assessment of potential lattice strains, and analysis and prediction of ionic migration paths within crystal structures.^11^ This theoretical framework offers several advantages that make it particularly suitable for our purposes. At its core, the bond valence theory allows for estimation of the bond order, the quantity reflecting the number of electron pairs shared between a given pair of atoms forming a bond. As eq 1 shows, the bond order or bond valence (*v*_*ij*_) calculation requires remarkably little input data: only the interatomic distance *r*_*ij*_ and tabulated parameters *R*_0_ and *b*, the latter assumed being transferable for a given atom pair *ij* across different chemical compounds.

1 $$v_{ij}=\exp\left[\left(R_{0}^{ij}-r_{ij}\right)/b_{ij}\right]$$

A particularly relevant advantage of the BV approach for our work is the well-known correlation between bond order and bond strength for many chemical bonds, where the larger bond order implies stronger bonding. Recent extensive research^12^ has demonstrated clear relationships between bond order and various descriptors of electron density topology, including electron density at the bond critical point ρ_c_, Laplacian of electron density, as well as strong correlations with the square of bond stretching frequencies. The relationships between ρ_c_(ij) and *v*_*ij*_ for a number of atom pairs are quasilinear for most of the pairs, while the slopes of the lines seem to agree with the type of interactions. These relationships provide a theoretical foundation that allows bond valence to serve as a **quantitative estimator of bond strength** for many types of chemical bonds. Consequently, bond order enables the creation of a new set of descriptors that can systematically characterize the distribution of bonding strength both within and between low-periodic substructures.

The bonding strength anisotropy descriptors have already been explored in several studies and demonstrated their utility through their successful application to the characterization of molecular crystal structures and mechanical properties. For instance, comprehensive analysis of intermolecular interaction dimensionality has been proposed and subsequently applied to comparative studies of chalcogen dicyanide series,^13^ detailed description of hydrogen-bonded guanidine structures,^14^ and the elucidation of bonding patterns in 2-bromomalonaldehyde crystals.^15^ This computational approach enables the decomposition of total lattice energy and attribution of the lattice energy fractions to low-periodic fragments (layers, chains, dimers, etc.) of the crystal, providing valuable insights into the main structural motifs of the crystal structure from an energetic perspective. Another illustrative example of an interaction anisotropy descriptor is the X parameter, specifically developed for identifying cleavage planes in molecular crystals.^16^

In this work, we develop a computational tool that addresses the limitations of current substructure search algorithms and applied it to expand the pool of exfoliable crystals. Specifically, we created an open-source *CrystalSubstructureSearcher* code capable of efficiently identifying low-periodic substructures within bulk crystal structures through a novel approach that leverages the bond valence theory for interatomic bond strength estimation. This approach combines the computational efficiency of simple interatomic distance-based methods with the quantitative descriptive power of bond valence theory. It enables rapid screening of large numbers of crystal structures and the analysis of sizable systems beyond the reach of DFT-based methods. Additionally, it provides valuable crystallochemical data, including substructure charge, proxies of substructure robustness, and descriptors characterizing bond strength anisotropy, useful for subsequent filtering. We benchmarked our method with literature results, other databases of low-periodic materials as well as dedicated ab initio simulations and then used it to study 48,504 experimental structures from the Inorganic Crystal Structure Database (ICSD)^17^ and Pearson’s Crystal Data (PCD)^18^ databases. Among them, we uncovered 694 previously unreported, robust, easily exfoliable materials that we later optimized and characterized in their isolated form with DFT simulations, with the exclusion of rare-earth-containing compounds.

## Results and Discussion

### Underlying Algorithm

The CrystalSubstructureSearcher is a code designed to analyze crystal structures and identify fragments or low-periodic substructures within them. Upon receiving a cif file with a crystal structure as input, the CrystalSubstructureSearcher initiates the analysis by constructing a graph representation of the crystal structure. The interatomic connectivity in the crystal structure is computed using the Voronoi algorithm as implemented in VoronoiNN class in *pymatgen*.^19^ The constructed graph, termed the structure graph (SG), consists of nodes representing atoms and edges representing bonds between atoms. A set of characteristics is computed for interatomic bonds, and then they are ascribed to each graph edge as attributes. The selected set of interatomic contact characteristics is meant to serve as a proxy of bond strength. It includes the interatomic distance (*R*); the area (*A*) and the solid angle (SA) of the Voronoi–Dirichlet polyhedron face corresponding to a given interatomic contact;^20^ the Penetration Index (PI) computed according to ref 21; the bond valence of the bond (BV) calculated using the bond–valence parameter *R*_0_. The *R*_0_ values for all 4851 atomic pairs of the first 98 chemical elements have been estimated by the ML regression model trained on the 874 empirical *R*_0_ values as described in the Methods section. For A, SA, PI, and BV descriptors, the larger values correspond to stronger bonds, while for *R*, inversely, smaller values correspond to strong interactions between atoms. The solid angle has already been applied^22^ as a bond strength criterion for the multilevel topological description of the molecular packings. One of the attributes from the set is selected by the user as the edge weight in the structure graph.

Extraction of the low-periodic substructures is the primary task of the implemented code. Identification of the substructures within the SG is done by the iterative removal of sets of SG edges with weights less than a threshold value. The threshold at each iteration increases progressively, starting from the smallest one if *A*, SA, PI, and BV are used as weights. On the contrary, if *R* is used as edge weight, the edge breaking starts from the largest values of interatomic distances. At each iteration, after a particular set of bonds has been broken, the dimensionality of the edited structure graph is checked. If the maximal periodicity of the SG components changed during the iteration, the graph obtained at that step is stored before proceeding further. Such an SG snapshot corresponds to the identified substructure with reduced periodicity, and it can be composed of several distinct SG components. For instance, two separate graphs could emerge that correspond to two distinct but possibly equivalent layers in the unit cell. One additional SG editing step is required after that, as in the preceding iterations bond sets corresponding to the contacts inside a component (*intra component contacts*) might be broken besides the *inter component contacts*. Therefore, an auxiliary procedure for bond set restorations is carried out. The bond sets recovered are those whose restoration does not lead to a change in the maximal periodicity of graph components. Overall, the described algorithm can be attributed to the class of greedy algorithms that solves its task by making locally optimal choices at each step. In our case, at each iteration, we break the weakest (as reflected by the selected edge weight) interaction. Though it does not guarantee to yield a globally optimal solution, in the case of the subgraph searching, it is the only viable method. The overall flowchart representing the algorithm is shown in Scheme S1 in the Supporting Information. Figure 1 schematically represents an analysis of the Ca_2_Sb structure.

**Figure 1:**
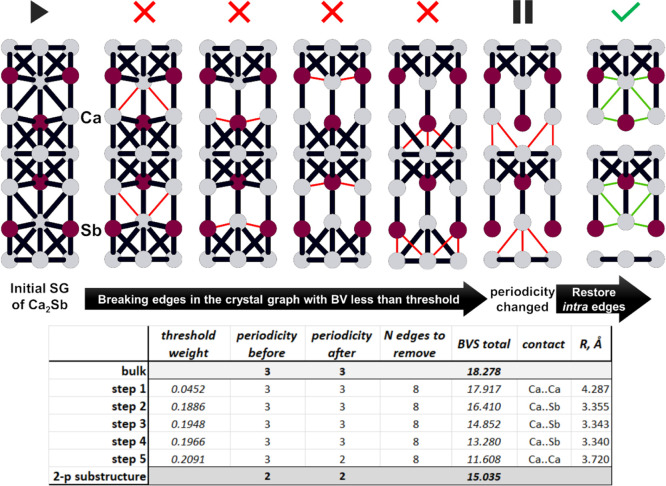
First iterations of the Ca_2_Sb structure (ICSD 154) analysis leading to identification of the 2-periodic substructure. The unit cell is shown down the *a* axis. The bond valences are selected as edge weight in this example. The edges of the SG broken at each iteration are highlighted in red, and their characteristics are shown in the table below. The edges restored at the final step are shown in green. *BVS total* is the sum of the bond valences of the retained edges in the SG components.

The algorithm terminates when the maximal periodicity of the substructures in the edited SG is zero. In other words, we stop at the point where all edges left correspond to internal bonds in the 0-periodic molecular fragments. As a result, the whole procedure yields a list of all of the low-periodic substructures of a given crystal structure. Next, the CrystalSubstructureSearcher computes for each substructure as well as for the initial structure graph a set of bond valence sum (BVS) descriptors that are meant to characterize the distribution of the bond strength in the crystal structure and across all of the substructures identified. The complete list of the computed BVS descriptors as well as other potentially useful data on the substructures and the interface between them is shown in Table S1.

Having computed the *BVS_x_periodicity* descriptor, which contains the data on the total BVS for each identified substructure, we can produce another important quantitative descriptor for the crystalline solids that reflects the bonding strength anisotropy. We call this characteristic *intrinsic solid periodicity*. It quantifies with a continuous value “how much” a crystal structure is 3-periodic, 2-periodic, or 1-periodic based on the strength of bonding between substructures of different periodicities, in contrast to traditional discrete periodicity classifications. In case of the Ca_2_Sb crystal structure, only 2-periodic intermediate substructure was identified; therefore, the total bond valence sums for the substructures collected in the *BVS_x_periodicity* descriptor are as follows: 3-periodic: 36.556 BV units, 2-periodic: 30.069 BV units, 0-periodic: 2.412 BV units. From these data, we can evaluate that the contacts that link up the layered Ca_2_Sb substructures into the bulk Ca_2_Sb solid have the strength of 36.556–30.069 = 6.487 BV units. Analogously, the interactions that combine the Ca atoms and CaSb dumbbells into the Ca_2_Sb layers amount to 30.069–2.412 = 27.657 BV units. Normalizing these BVSs attributed to the contacts that “build up” a given periodicity substructure, we obtain the sought after descriptor of the intrinsic solid periodicity. In the case of Ca_2_Sb crystal structure, the largest intrinsic periodicity is found in the 2-periodic substructure. Therefore, most of the bonding is confined between building units (Ca atoms and CaSb molecular fragments) that form the layers (*2-p*: 0.81) rather than between the layers themselves, which link together to form a bulk crystal (*3-p*: 0.19).

In addition to substructure identification and BVS descriptor calculation, the CrystalSubstructureSearcher is capable of estimating substructure charges. This process involves calculation of the electronegativity difference and bond valence of the contacts between SG components, providing a valuable estimation of the charge distribution over the identified substructures. In summary, the CrystalSubstructureSearcher employs a step-by-step algorithmic approach to analyze crystal structures, encompassing low-periodic substructure identification, calculation of BVS descriptors and intrinsic periodicity, interface characteristics, and substructure charge estimation.

### Algorithm Validation and Correlation between Ab Initio Interlayer Binding Energy and BVS-Based Descriptors

To benchmark and calibrate our method, we first ran a search for 2-periodic substructures on a data set of 1713 theoretically optimized bulk crystal structures, originally used to create part of the MC2D database.^1,2^ This data set includes structures previously classified as containing layered substructures that are either easily exfoliable (784 structures with interlayer binding energies not exceeding 30 meV/Å^2^) or potentially exfoliable (929 structures with binding energies between 30 and 120 meV/Å^2^). The algorithm implemented in CrystalSubstructureSearcher has failed to identify the two-periodic substructures in only two structures from the easily exfoliable subset and in 26 from the potentially exfoliable subset (see the *no_layers_identified* sheet in the Supporting Information.xlsx file). Further, only for 52 structures (see the *different_layers_identified* sheet in the Supporting Information.xlsx file), the layers identified with new algorithm were different from those extracted previously using the distance-based algorithm.^1^ Therefore, the new algorithm has proven to produce meaningful results coherent with the distance-based algorithm, as for 1633 out of 1713 (95.3%) structures identified, substructures are the same. The overwhelming majority of the 2-periodic substructures identified in the structures from the easily exfoliable subset (775 structures) were neutral as anticipated for the solids with weak vdW interactions on the interface between layers. Charged layers have been found in only two structures, though with the negligible estimated charges not exceeding 0.26|*q*_*e*_|. On the contrary, for 30 structures from the potentially exfoliable subset (858 structures), charged low-periodic substructures were found.

The most appropriate way to visualize the intrinsic periodicity is to use a ternary plot since all the intrinsic periodicity values sum up to 1.0. Such a plot representing the position of crystal structures from the easily exfoliable subset in the intrinsic periodicity coordinates is shown in Figure 2a. As expected, only a few points lie close to the triangle corner corresponding to the intrinsically 3-periodic crystals, with the mercury­(I) nitrite being the closest to the intrinsically 3-p solids (*3-p*: 0.67, *2-p*: 0.33). In total, 65% of the structures in this subset have the share of intrinsic 2-p character 2-p ≥ 0.5, i.e., these are truly intrinsically 2-periodic crystals. Besides the 2-p corner, the structures concentrate also near the midpoint of the 1-p/2-p side, and to a lesser extent close to the 1-p corner. These structures correspond to layered structures with layers formed by progressively larger rods with more and more interatomic binding concentrated inside rods than between them. This is exemplified by the SiP structure (*3-p*: 0.007, *2-p*: 0.049, *1-p*: 0.945), which has the largest 1-p intrinsic periodicity. Comparison with the potentially exfoliable subset plotted in Figure 2b shows that the distribution of points is shifted out of the 1-p/2-p side toward the center of the triangle, which is rather anticipated for the structures with less pronounced layered character and higher interlayer binding energy values. Overall, this descriptor of bonding anisotropy is a valuable tool for distinguishing the stable layered motifs from rod-like layered structures that are prone to disintegration into 1-periodic fragments.

**Figure 2:**
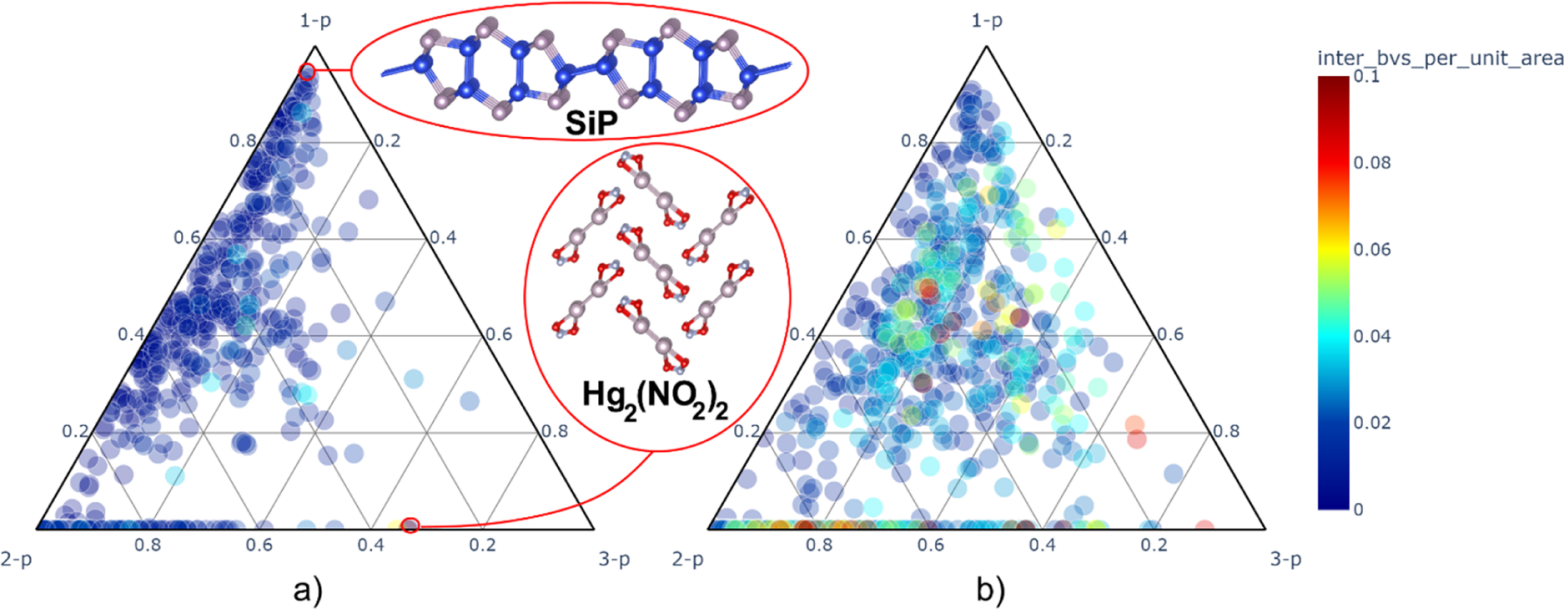
Representation of the intrinsic periodicity of the crystal structures in the data set used for the MC2D database creation. (a) Ternary plot showing 782 structures from the easily exfoliable subset (*E*_binding_ less than 30 meV/Å^2^) in the intrinsic periodicity coordinates. (b) The same plot for 903 structures in the potentially exfoliable subset (*E*_binding_ in the range from 30 to 120 meV/Å^2^). The points are colored according to the *inter_bvs_per_unit_area* descriptor values.

A moderate overall correlation (Spearman’s rank-correlation ρ = 0.83, Pearson’s *r* = 0.58) was found between binding energy per unit area (*E*_bind_/*A*) and bond valence sum per unit area (BVS/*A*) descriptors (Figure S1a), which still is quite good, especially taking into account that the interfacial bonds of 401 different contact types and 754 different contact types combinations are shown in the scatterplot (Figure S1b). However, if we “zoom in” and consider correlations between *E*_bind_/*A* and BVS/*A* for particular bond types, we can find much stronger correlations. For instance, in Figure S2, the scatterplots show higher correlation coefficients (ρ = 0.69 ÷ 0.96) for homoatomic pnictogen (Pn···Pn), chalcogen (Ch···Ch), and halogen (Hal···Hal) contacts formed by atoms from the same period. The analysis of the structures with abundant interlayer contact types (at least 5 structures) suggests that in principle, different threshold BVS/*A* values should correspond to different contacts because of the different *E*_bind_/*A* vs BVS/*A* dependencies (Table S2). Unfortunately, only for 26 contact types encountered in 615 structures (37.7%) did the OLS fitting of the *E*_bind_/*A* vs BVS/*A* linear relationships result in statistically significant (at α = 0.05) slope coefficients. Most of the interlayer contact types and their combinations (Figure S1b) are encountered in only 1–3 structures, which obviously renders impossible obtaining reliable dependencies from the OLS fitting. Therefore, we needed to devise an approach that would expand applicability of the algorithm for larger sets of structures with variety of the contact types on the interface. We came up with a simplistic approach and decided to “aggregate” the atoms by grouping them such that atoms with similar properties end up in the same group. For instance, the alkaline and alkaline-earth metals are collectively designated as “electropositive metals” (EPMs). We set up nine coarse groups of atoms according to their similarity, as shown in Table S3. Then, we simply mapped the atoms forming interfacial contacts to these aggregated atom groups and obtained new aggregated interfacial contact types. For example, both Cl···Cl and Br···Br contacts were mapped to the NM···NM (non-metal) contact group, while Cl···Cl|Cl···Sr and Br···Br|Br···Ca were mapped to the corresponding EPM..NM|NM..NM contact group. In total, 263 different combinations of such aggregated contact groups were obtained, an almost 3-fold decrease compared to 754 initial contact types. After that, we used the contact groups encountered in at least 7 representative structures to model the *E*_bind_/*A* vs BVS/*A* linear relationships using the Robust Linear Model (RLM) as implemented in *statsmodel* library.^23^ The RLM model with the Huber loss function was used for fitting because it gives more stable regression coefficient estimates in the presence of large outliers, which emerge after contact grouping. As a result, for 29 aggregated contact types covering 1119 structures (68.5%), we obtained line fits with statistically significant regression coefficients (Figure S3), which is an almost 2-fold improvement compared to plain contact types. Finally, using the obtained regression coefficients, we calculated for each aggregated contact type the threshold value of BVS/*A* that corresponds to 30 meV/Å^2^ threshold binding energy (see Table S4 in the Supporting Information). In addition, we increased the BVS/*A* thresholds by 20% so that more structures on the verge of the arbitrarily selected borderline of 30 meV/Å^2^ could be accepted. The proposed grouping strategy might smear out a certain degree of chemical difference between the representatives of the same group, but we estimated the associated error to be relatively small as discussed in the Supporting Information.

To validate the obtained *E*_bind_/*A* vs BVS/*A* dependencies, we utilized a new set of crystal structures with layered substructures taken from ICSD characterized by a small-enough unit cell to be readily treated with DFT calculations (up to 12 atoms) and not present among the structures used to create the MC2D database (that used structures from ICSD version 2018). The test set comprised 162 structures with computed values below 120 meV/Å^2^. The binding energy calculations were performed using the same procedure employed in the creation of the MC2D database, as described in ref 1 and 2 . Among these crystal structures, only 95 contained interlayer contact types that belong to one of the 29 aggregated contact types, enabling *E*_binding_ estimation using BVS/*A* descriptors (see the *Ebinding_calc_vs_estimated* sheet in the Supporting Information.xlsx file). The scatterplot in Figure 3 demonstrates that simple linear relationships for aggregated contact types provide binding energy estimates sufficiently accurate for screening purposes. Notably, the regression model’s tendency to overestimate *E*_binding_ ensures conservative screening by minimizing false positives: hard-to-exfoliate materials will not pass, though some exfoliable structures may be excluded. The mean absolute deviation is only around 10 meV/Å^2^, confirming the effectiveness of such a simple and straightforward approach.

**Figure 3:**
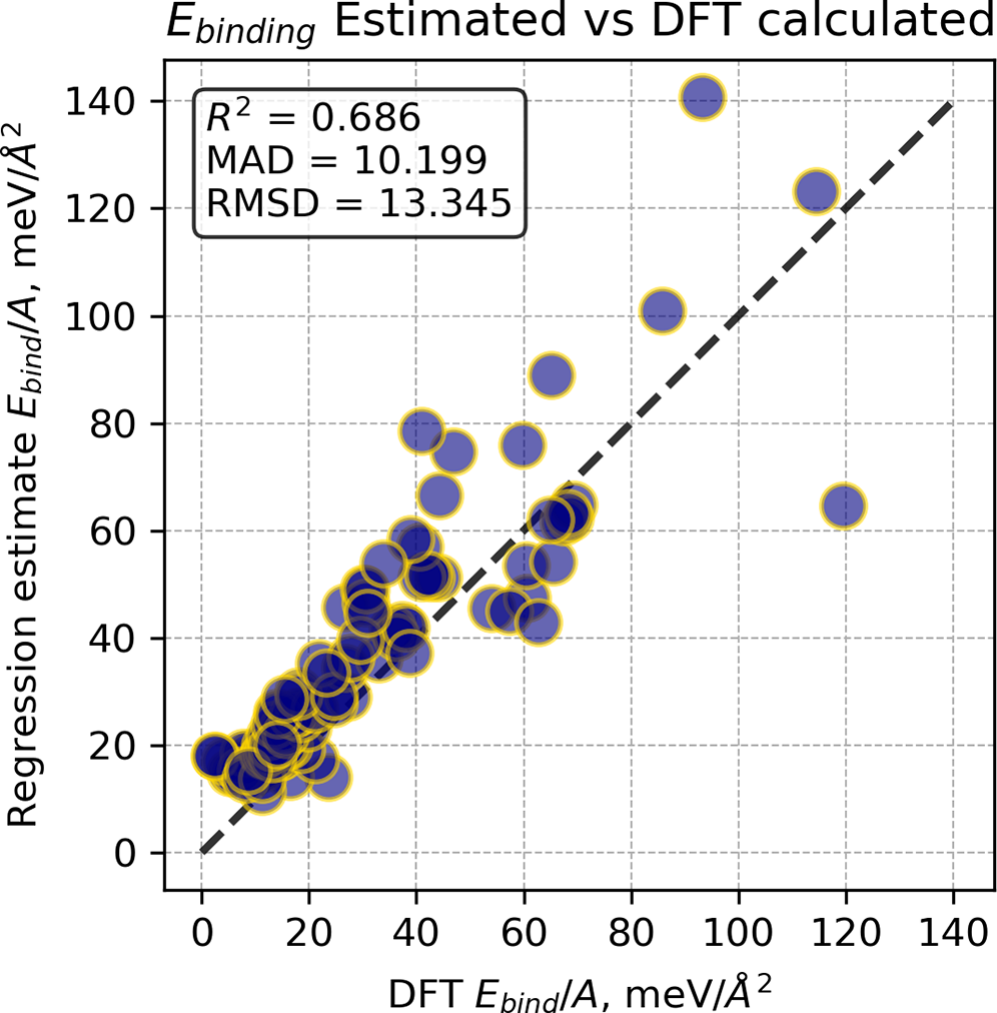
Comparison of the *E*_binding_ calculated using DFT methods with binding energy estimates by means of linear *E*_bind_/*A* vs BVS/*A* relationships in a test set of 95 structures.

To verify whether our method can be reliably employed directly on experimental data without the need of an intermediate and potentially expensive optimization step, we have also analyzed with CrystalSubstructureSearcher the experimental crystal structures used for the creation of the MC2D database as taken directly from the crystallographic databases without any DFT optimization of the bulk cell geometry. The relative percentage differences of the BVS/*A* obtained for DFT-optimized structures relative to the experimental crystal structures are shown in Figure S4 in the Supporting Information. Overall, DFT optimization leads to an increase of the BVS/*A* descriptor as observed in 72% of structures, due to shortening of the interlayer contacts. The absolute percentage difference between values of BVS per unit area descriptor optimized with respect to original structures does not exceed 25% in 56.1% of structures.

### ICSD Filtering and Program Application on a Large Set of Structures

After developing the algorithm, implementing it in the CrystalSubstructureSearcher program, and validating its performance on a data set with known interlayer binding energies, we proceeded to apply it to much larger structure sets to demonstrate its capacity of identifying crystal structures with novel low-periodic substructures. The crystal structure data from the ICSD^17^ and PCD^18^ databases underwent a series of filtering steps, designed to ensure the selection of only the highest-quality structures. As a result, the final data set contains 48,504 inorganic compounds analyzed with the CrystalSubstructureSearcher code. The analysis of the data set with CrystalSubstructureSearcher was aborted for 1390 structures (2.9%), the main reasons being too short interatomic distances, errors during the Voronoi polyhedra construction, and other structural abnormalities and peculiarities. In the set of 47,114 crystal structures for which analysis finished successfully, 2-periodic substructures were identified in 27,350 structures (Figure 4a). Ternary plot in Figure 4b shows the distribution of intrinsic periodicity of the structures. The crystal structures in which both 1-p and 2-p substructures exist accumulate close to the triangle center. The structures with only 1-p substructures amass closer to the 3-p vertex of the triangle, that is most of them have larger 3-p character than the 1-p one. On the contrary, the structures with only 2-p substructures concentrate closer to the 2-p vertex of the 2-p/3-p triangle side. Overall, we can see that the structures from the easily exfoliable subset discussed previously are located in the sparsely populated area of the ternary plot (cf. Figure 2a).

**Figure 4:**
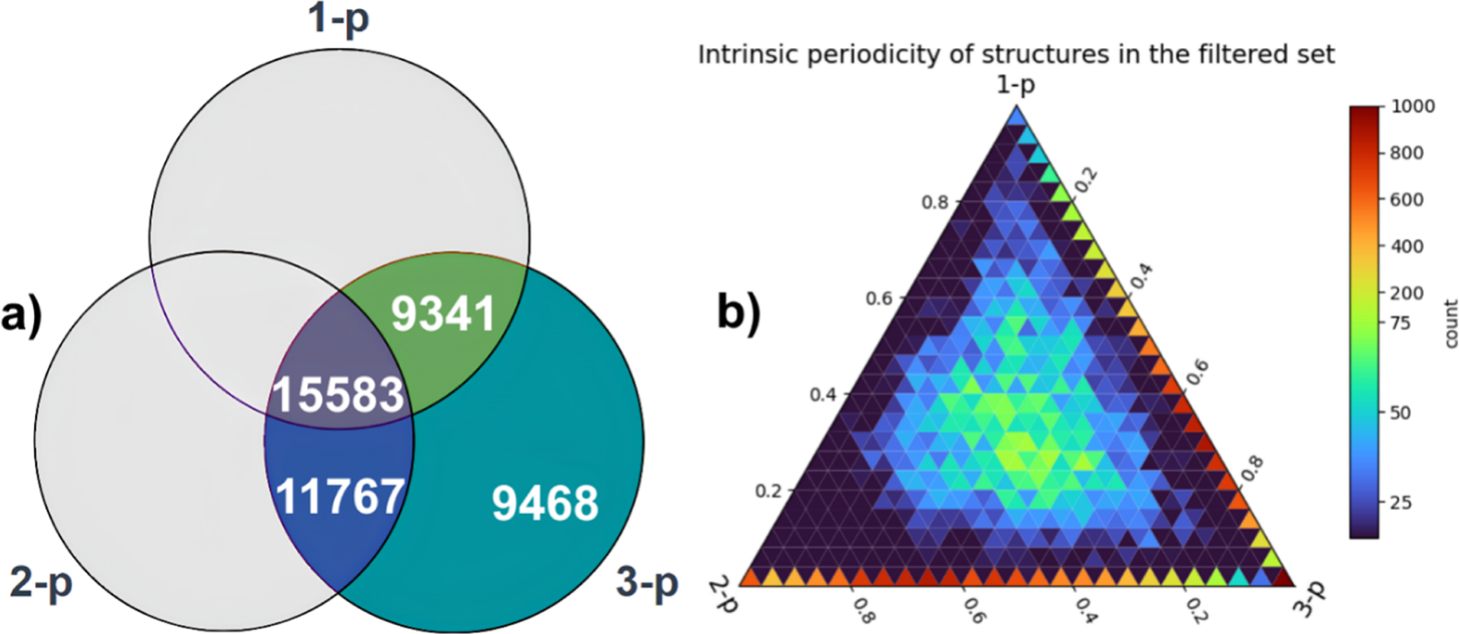
(a) Venn diagram reporting the counts of crystal structures in which low-periodic substructures are present. Structures in which substructures of all periodicities have been found correspond to 34% of all the structures analyzed, structures with only 2-p substructure to 25%, structures with only 1-p substructure to 20%, and the rest 21% have no low-periodic substructures. (b) Ternary plot showing the structures filtered from the ICSD in the intrinsic periodicity coordinates. Note the 9468 structures without low-periodic substructures located in the 3-p corner of the triangle. For 955 structures, calculation of the intrinsic periodicity was not possible due to the structure peculiarities.

The identified threshold values of the BVS/*A* descriptor for 29 aggregated contact types allowed us to estimate exfoliability of only 8343 structures (out of 27,350), 2135 of which had BVS per unit area lower than the threshold corresponding to 30 meV/Å^2^; thus, we expect that these structures contain weakly bonded layered substructures. Additionally, we have filtered out structures with charged substructures and finally we have got a set of 1989 potentially easily exfoliable crystal structures with neutral substructures (estimated charge <0.25|*q*_*e*_|, the value chosen is close to the maximal absolute charges of the layers identified in the MC2D easily exfoliable subset as discussed previously). Finally, we compared the list of identified substructures with existing databases to assess the novelty of our results (hereinafter, CSS results). For the comparison, we used latest available versions (as of June 2025) of MC2D^1,2^ (https://mc2d.materialscloud.org), 2DMatpedia^3^ (http://www.2dmatpedia.org), and C2DB^4^ (https://c2db.fysik.dtu.dk) databases containing results of the application of distance-based algorithms to large sets of both experimental and theoretical crystal structures from ICSD, COD, and Materials Project databases. Importantly, besides the theoretically exfoliated layers (the top-down approach), the 2DMatpedia and C2DB databases also contain the two-periodic substructures created via the bottom-up approach, in which elemental substitution is systematically applied to the layers identified with the common top-down approach. To avoid false positives in the identification of novel structures potentially arising from the differences in the structural optimization procedures used in different databases, we opted for a more conservative composition-based comparison. We compared the compositions of the 2-p substructures identified via the top-down approach stored in the three databases and those identified by CrystalSubstructureSearcher. As shown in the Venn diagram in Figure 5a, 929 unique layer compositions (identified in 963 crystal structures) absent from the other databases of 2D materials have been identified by CrystalSubstructureSearcher. One more filtration step is required to get the final shortlist of the robust layer. As was noted in ref 6, “···if the in-plane strength of a layered crystal is not strong enough to resist the interlayer interaction, the material’s plane will be fractured during the mechanical exfoliation, and hence this layered crystal is not an exfoliable crystal”. To address this challenge and to eliminate fragile layers from the set, an additional BV-based descriptor, designated as *max_intracomponent_bond_strength*, was devised. This descriptor reflects the strength of the bonds through which the 1-p or 0-p substructures are held together to form a 2-p substructure. Examples of application of this descriptor are given in Figure 5b. The layer extracted from the W_5_I_11_ crystal structure is built from tungsten clusters connected through strong W–I bonds (0.57 BV units), and it is precisely this bond that imparts the robustness to the layer. On the contrary, 2-p substructure extracted from Cu­(SCN)_2_ is composed of chains connected through weak Cu–S bonds (0.05 BV units). Consequently, it is probable that such layer will fracture upon exfoliation. To identify the threshold value of the *max_intracomponent_bond_strength* descriptor, we checked the corresponding distribution in the set of 1633 easily exfoliable materials from MC2D. Given that 99% of layers in the data set have a maximum intracomponent bond strength greater than 0.1 BV unit with a distinct slope change in the distribution around this value, this value was set as threshold. The distributions for the *max_intracomponent_bond_strength* descriptor in MC2D and the CSS results data set are reported in Supporting Information.

**Figure 5:**
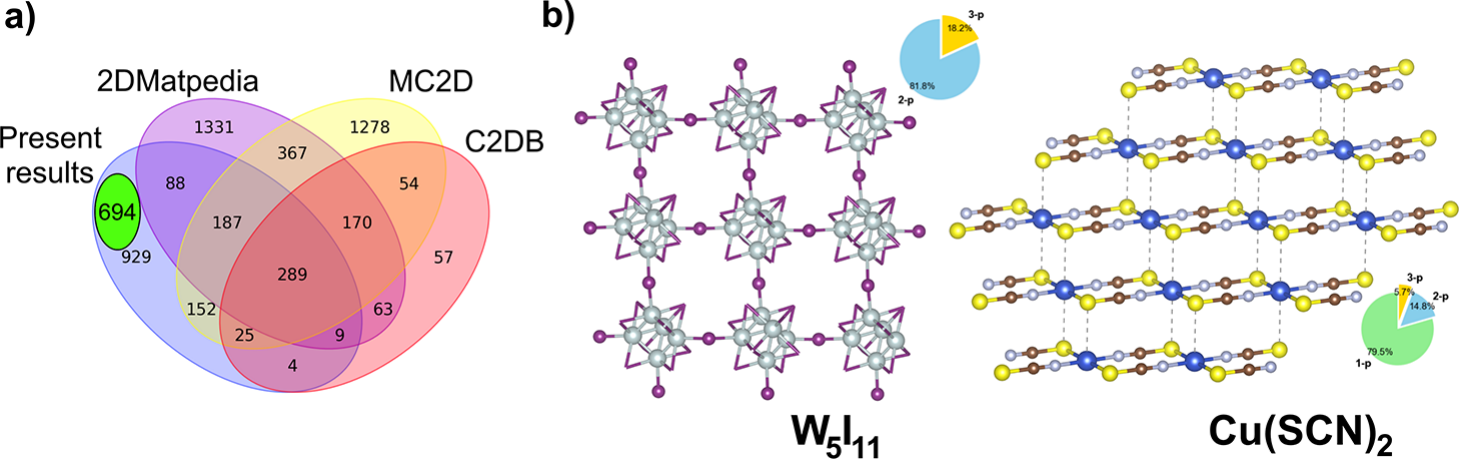
(a) Venn diagram showing the intersections of the compositions of layers in the four sources. Note that only layers obtained from the crystal structures—so-called top-down approach—are taken into account. Total counts of unique layer compositions in each data set considered are as follows: CSS results—1683 compositions, 2DMatpedia—2504 compositions; MC2D—2522 compositions; C2DB—671 compositions. Additionally, a subset of 694 robust layers is shown. (b) Examples of the structures in the *new_prospective_exfoliable* subset with more pronounced 2-p character (W_5_I_11_, ICSD 244435, oP64) and predominantly 1-p crystal structure (Cu­(SCN)_2_, ICSD 131336, aP7) with weak interchain Cu..S contacts inside the identified 2-p substructure. The intrinsic periodicity is shown as pie chart.

Filtering the list of 929 unique layer compositions absent from other databases, we obtained a shortlist of 694 robust layers (see the *new_prospective_exfoliable_shortlist* sheet in the Supporting Information.xlsx file) found in 712 crystal structures. These robust layers are distributed over 530 different structural prototypes, crucially expanding by almost 40% the number of possible structural templates with respect to previous works,^2^ thus opening a significant part of the materials space to future theoretical exploration based on elemental substitution. The majority of such newly identified materials is represented by layers derived from bulk structures with large unit cells that have been excluded from previous studies due to their prohibitive computational cost. Another significant fraction is represented by materials derived from structures only recently deposited in the experimental databases. Interestingly, the analysis of the shortlist revealed layered structures that have never been captured by the previous algorithms. For instance, the neatly layered compounds Bi_3_Te_2_S (ICSD 107587, hP12), Gd_2_Te_5_ (ICSD 636468, oS28), as well as less evidently layered ones like EuI_2_ (ICSD 260561, oP12), Cs_3_Bi_2_I_9_ (ICSD 410726, hP28), or Cs_2_C_4_O_4_ (ICSD 154357, mS20) were not deposited in any of the 2D materials databases quoted above.

The ultimate confirmation of the algorithm’s validity and generality is demonstrated by its successful identification of a recently emerged group of nonvan der Waals 2D materials, which were reported in two recent publications.^24,25^ In these studies, exfoliation energies were calculated for a set of relaxed structures, primarily mixed metal oxides, as well as NaMnCl_3_, Al_2_S_3_, and In_2_S_3_—all belonging to the corundum structure type. All in all, 30 out of 36 compounds mentioned in the studies appeared in the filtered ICSD subset referenced earlier and were analyzed using the CrystalSubstructureSearcher algorithm (see the *non_vdW_2D* sheet in the Supporting Information.xlsx file). Among these, neutral two-periodic substructures were successfully identified in 27 compounds. For NaSbO_3_ and KSbO_3_, the algorithm detected negatively charged SbO_3_ layers accompanied by alkali metal cations. Instead, for Rh_2_O_3_, no layered substructure was found due to the negligible difference in the two sets of Rh–O distances.

### Ab Initio Characterization of the Robust Layers

As a final step, to offer an overview on the properties of the extracted layers, we optimized and characterized with ab initio methods 550 extracted robust layers excluding rare-earth-containing compounds that are notoriously difficult to properly describe in plain DFT due to the localized *f*-orbitals. The computational details are reported in the Methods section. Due to the reduced size of the layers with respect to the parent bulk material as well as the reduced dimensionality, this characterization effort is an order of magnitude less computationally expensive than a full ab initio study of the parent materials. In Figure 6, we reported the root-mean-square displacement of the scaled atomic coordinates and the variation of the 2D unit cell surface between the as-extracted layer and the theoretically optimized one. These two quantities can be used as a qualitative estimation of stability of the extracted layers since small variations are expected for materials prone to exfoliation in which neither the internal atomic arrangement nor the cell is expected to change dramatically between bulk and the isolated layer. Interestingly, the values found for the successfully optimized materials are comparable to what was observed for the materials in the MC2D database,^1,2^ further hinting on the high quality of the extracted layers.

**Figure 6:**
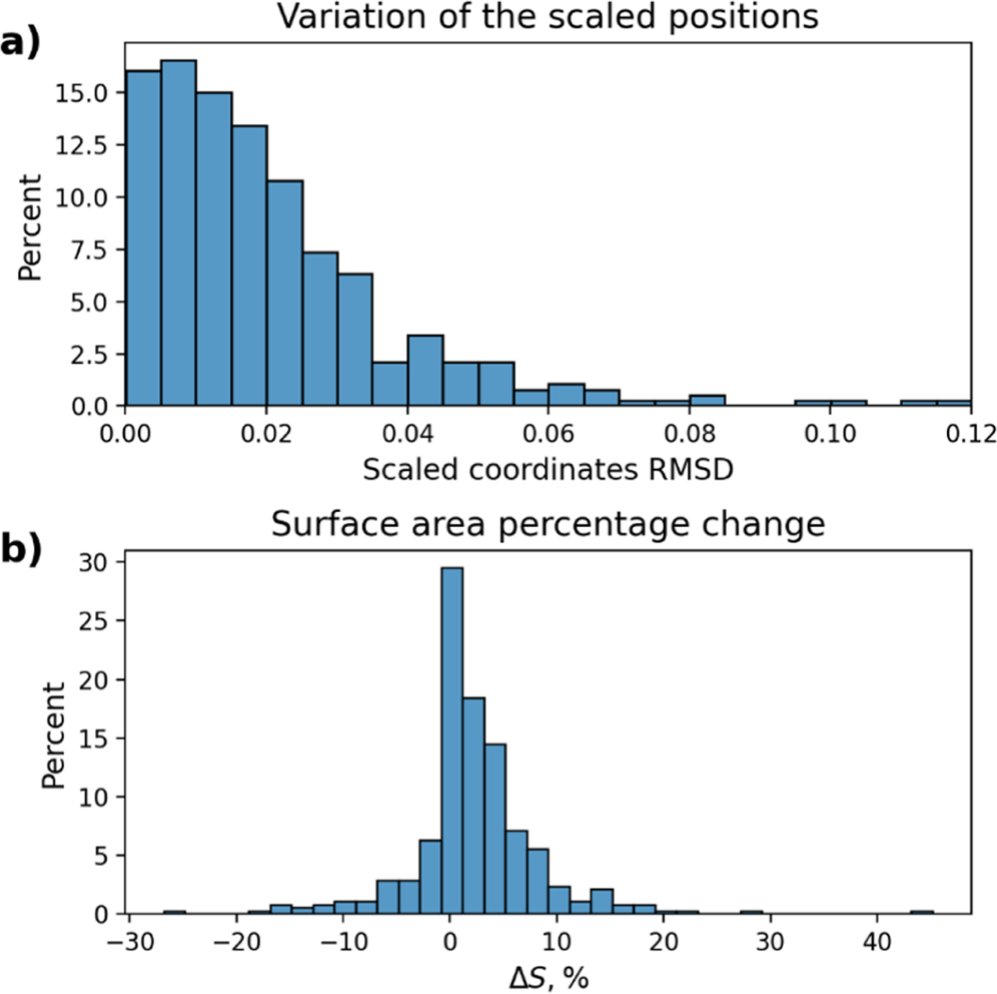
(a) Distribution of the root-mean-square displacement of the scaled atomic coordinates between the as-extracted and theoretically optimized layers. (b) Distribution of the unit cell surface variation between the as-extracted and optimized layer.

Finally, we performed a basic characterization of the electronic properties, computing the band structure along the proper 2D high-symmetry path for the optimized layers. The full band structures are reported in the Supporting Information, while the distribution of the bandgaps for the semiconducting and insulating compounds is reported in Figure 7. With respect to the results obtained in ref 2, the distribution peak is shifted toward higher bandgaps (around 2 eV at the DFT level) in a region particularly promising for photochemistry, transparent, and semitransparent electronics, exciton-based devices with the possibility to realize stable excitons at room temperature. It is also worth noting the presence of several candidates in the scarcely populated region of high bandgaps (above 6 eV at the DFT level) that can be crucial for high-performance dielectric materials.

**Figure 7:**
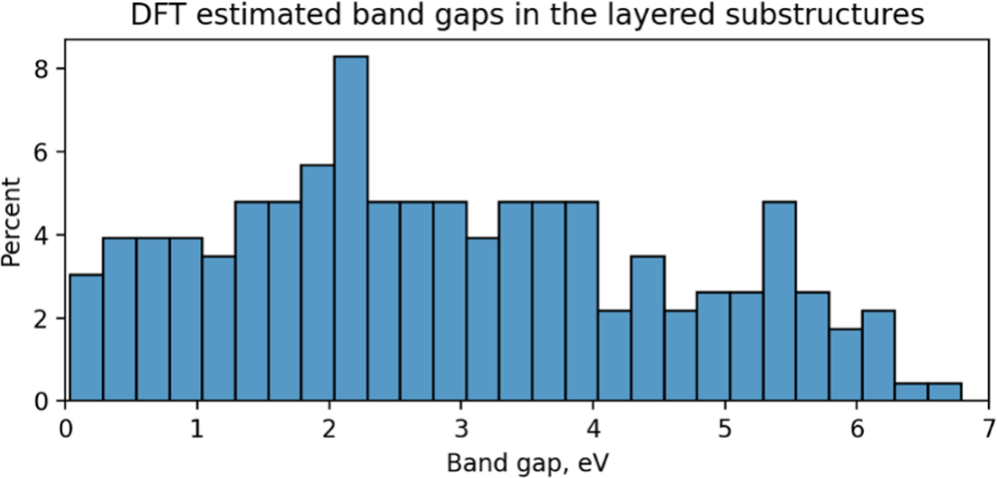
Distribution of the fundamental bandgap at the DFT level for the insulating and semiconducting 2D materials.

## Conclusions

In this work, we developed, benchmarked, and applied a flexible and computationally efficient algorithm to identify low-periodic substructures in bulk crystals and assess their exfoliation ease. The algorithm is implemented in the open-source CrystalSubstructureSearcher code and operates by constructing a graph representation of the crystal structure with edges weighted by descriptors reflecting the bonding strength. Low-periodic substructures are identified through the iterative removal of the weakest links while monitoring the dimensionality of the resulting substructure. The algorithm was validated on a data set of structures from the MC2D database for which 2-periodic substructures were identified with distance-based algorithms and DFT-estimated interlayer binding energies were available. This comparison also allowed us to design an efficient strategy to correlate the DFT-derived binding energies with the BVS per unit area descriptor for a large number of contact types, enabling the possibility of a rapid approximate estimation of the exfoliation energy without the need for direct DFT calculations. We further applied such methodology to a data set of 48,504 entries filtered from the ICSD and PCD databases including large structures that have been discarded in previous studies as being too expensive for direct high-throughput DFT searches. This allowed us to identify 694 robust, easily exfoliable two-dimensional materials whose compositions are not present in current 2D materials databases (MC2D, C2DB, and 2DMatpedia), demonstrating the potential to significantly expand the known 2D-materials space, particularly toward large unit cell systems that are challenging for DFT-based approaches. In addition, we have proposed a crystal intrinsic periodicity concept and the corresponding descriptor that allows for characterization of the bonding anisotropy in crystals. The descriptor quantitatively reflects the 1-p/2-p/3-p character of the crystal structure. As a final step, we optimized and computed the band structures for a large portion of the newly identified 2D materials, offering a coarse overview of their electronic properties that can help guide future application-oriented screenings. As a collateral outcome, we have obtained estimates for *R*_0_ BV parameters for all possible bonds between the first 98 elements. The predicted *R*_0_ values were compared against the ground truth *R*_0_ values in the test set with excellent resulting regressor’s performance as reflected by the RMSE (0.034 Å), *R* ^2^ (0.965), and MAE (0.026 Å) metrics. The new set of *R*_0_ BV parameters can be employed in bond valence sum mapping methods used for visualizing mobile ion diffusion pathways within the crystal structures of solid electrolytes.^26^

## Methods

### Extension of the Bond Valence Parameters Set Using the ML Regression Model

The BV parameters *R*_0_ and *b* are fitted commonly by using the experimental crystal structure data such that the bond valence sum around a given atom should be equal to its valence state, which is usually set manually for each atom in the structure. This means that the fitting of the bond valence parameters in crystal chemistry is based on the formal values of shared electrons. Though both BV parameters could be fit, usually only the *R*_0_ parameter is fit, while constraining the *b* parameter to a fixed value of 0.37. Nevertheless, still not for all bonds BV parameters are available. The comprehensive list of the bond valence parameters available at the moment was taken from the dedicated IUCr web-page.^27^ However, in this source, the BV parameters are present only for 889 bonds with *b* = 0.37; thus, empirical data is limited particularly for less common atomic pairs or pairs of metals that nevertheless might be encountered during the analysis of crystal structures in crystallographic databases. To address this challenge, we decided to employ a machine learning (ML) approach to estimate the *R*_0_ parameter for all 4851 atomic pairs of the first 98 chemical elements. Our approach followed a strategy similar to that proposed in ref 28 where the unknown *R*_0_ parameter was approximated as the sum of respective element size parameters *r* adjusted by a correction term (Δ_*ij*_) that comprises the second *c* parameter correlated with the electronegativity (EN) of the atoms (eq 2). The *b* parameter in the study has been fixed at 0.37, a common choice in the estimation of BV parameters, though it has been shown that optimizing also the *b* parameter might be beneficial and result in better modeling of the bond order in ref 10,29 .

2 $${R_{0}^{ij}=r}_{i}+r_{j}-\Delta_{ij}=r_{i}+r_{j}-\frac{r_{i}r_{j}{\left(\sqrt{c_{i}}-\sqrt{c_{j}}\right)}^{2}}{\left(c_{i}r_{i}+c_{j}r_{j}\right)}$$

3 $${R_{0}^{ij}=r}_{covi}+r_{covj}-\Delta_{ij},,where\;\Delta_{ij}=f\left(EN,Z_{eff},period,vdw\text{\_}crust\text{\_}V\text{\_}portion\right)$$

In a similar manner, we have fixed the *r* size parameters to be equal to the element covalent radii^30^ and have fixed the *b* parameter value to 0.37 so that the regression model has been trained to estimate the correction factor Δ using a set of atomic properties taken from the *mendeleev* python library^31^ as independent variables (eq 3). In principle, a multitarget regression model could be used to obtain an improved set of both *R*_0_ and *b* BV parameters, but this would be the topic of future studies. Here, we focused on testing the crystal structure analysis algorithm and on extending applicability of the BV theory exploited in it to all compounds in the ICSD.

The *R*_0_ parameters in the fetched IUCr data depend also on the oxidation and spin states of atoms forming a bond, though this dependence is only marginal in most cases which is also supported by the earlier study.^32^ Nevertheless, relatively large ranges of tabulated *R*_0_ values are observed for bonds between transition metals and O/N/C atoms and for Tl–X bonds. We decided to neglect this dependence when training the ML model, since this would allow us to analyze the crystal structures without additional data on oxidation number of the elements and their spin state. This is important, because many crystal structures lack this kind of information, or it may be hard to assign a specific oxidation state to the atoms in the structure as exemplified by copper chalcogenides.^33^ Thus, for the atomic pairs with multiple *R*_0_ values, the target *R*_0_ value is obtained by averaging the empirical *R*_0_ parameters. In total, this approach yielded a data set of empirical *R*_0_ parameters for 874 bonds comprising 90 elements. Parameters marked as “unchecked” in the original database were excluded as unreliable. Figure S5 in the Supporting Information shows for each separate bond type the share of bonds for which the empirical *R*_0_ parameters exist. Bond types for which there are sufficient empirical data (we set a limit of 20% of all possible atomic pairs in the group) have reliable model estimates of *R*_0_. For other bond types with less than 20% empirical data on atomic pairs, the model estimates of *R*_0_ are considered unreliable. The proportion of bonds with unreliable ML-estimated *R*_0_ parameters is recorded for each structure analyzed.

After collecting, processing, and preparing the BV parameters data, we have created informative atomic pair feature representation for subsequent model training. The selected set of 8 atomic properties has been used to obtain the description of each atom forming a bond. The set includes five different EN scales EN_Rahm-Hoffmann,^34^ EN_Allred-Rochow,^35^ EN_Nagle,^36^ EN_Martynov-Batsanov,^37^ effective nuclear charge Z_eff_, number of period, and vdw_crust_V_portion, calculated as $1-{\left(r_{cov}/r_{vdw}\right)}^{3}$ and reflecting the portion of the atomic volume occupied by the vdW crust.^21^ The recent set of the elements vdW radii was employed.^38^ Since the two sets of atomic descriptors are produced for each atomic pair, the order in which the features are composed does matter. To resolve the ordering problem, the atoms in each atomic pair have been sorted according to their EN in the Allred-Rochow EN scale; the descriptors of the atom with smaller EN come first in the resulting concatenated vector.

The CatBoost gradient-boosted decision trees regression model^39^ was chosen as an ML algorithm for the task of predicting the Δ_*ij*_ correction factor in eq 3. The algorithm iteratively adds the decision trees to the ensemble, minimizing the RMSE loss function and gradually improving its predictive accuracy. The Δ_*ij*_ values were obtained by subtraction of the *R*_0_ values from the sum of covalent radii of atoms (the distribution of the target Δ_*ij*_ values is shown as the histogram on Figure S6 in the Supporting Information). For the feature representation of bonds, a variety of techniques have been tested to identify the best combination mode of the separate atomic features, including averaging of atomic features, calculations of their differences, and division, as well as simple concatenation of atomic feature vectors. The 5-fold cross-validation has been used to explore different feature engineering strategies by training the regressor with a predefined set of hyperparameters (Table S5 in the Supporting Information). As a result, the simplest representation of atomic pairs as a concatenation of atomic feature sets showed the lowest RMSE in the task of predicting the correction factor Δ_*ij*_ (Figure S7 in Supporting Information).

Further, after choosing the feature representation of bonds, a simple regressor training strategy has been applied. The data set was split into three parts with 655 entries in the training set, 120 in the evaluation set, and 99 in the test set. The early stopping with a patience of 10 rounds was used to prevent overfitting of the gradient boosting regression model and selection of the optimal number of decision trees in the ensemble. The model was then trained with a fixed learning rate of 0.05 on the training set while monitoring its performance on the evaluation set. As a result, the best ensemble comprising 399 trees was used to make predictions for the test set. The estimated *R*_0_ values were compared against the ground truth empirical *R*_0_ values in the test set to evaluate the regressor’s performance using RMSE (0.034 Å), *R* ^2^ (0.965), and MAE (0.026 Å) metrics. Finally, the model with the optimal hyperparameters was retrained on the whole data set of 874 entries and further was used to estimate the correction factor Δ_*ij*_ for the set of all 4851 atomic pairs in the set of 98 elements (see the *ML_BV_parameters* sheet in the Supporting Information.xlsx file). The overall accuracy of the model predictions compared with the empirical data on 874 bonds is shown in Figure S8 in the Supporting Information and in Figure S9 for separate bond groups.

### ICSD and PCD Data Set Filtering

The crystal structure data were taken from the ICSD^17^ (version 2023/2) and PCD^18^ (version 2014/15) databases and underwent a series of filtering steps concisely shown on the scheme in Figure 8. Structures in the filtered set were grouped based on their chemical formulas, and subsequently, the structural similarity has been estimated using a *StructureMatcher* algorithm as implemented in *pymatgen*. As a result, for each group of compounds with the same formulas, a set of subgroups was identified, each of which contained duplicated crystal structures—different structure solutions of a distinct crystalline compound or its specific polymorph. The single representative structure within groups containing several structure determinations was selected based on the following criteria: if the *R*_f_ values were reported for the database entries, the one with the lowest *R*_f_ has been selected; if the *R*_f_ values were not available for all database entries in the group, the most recent (by publication year) structure was chosen.

**Figure 8:**
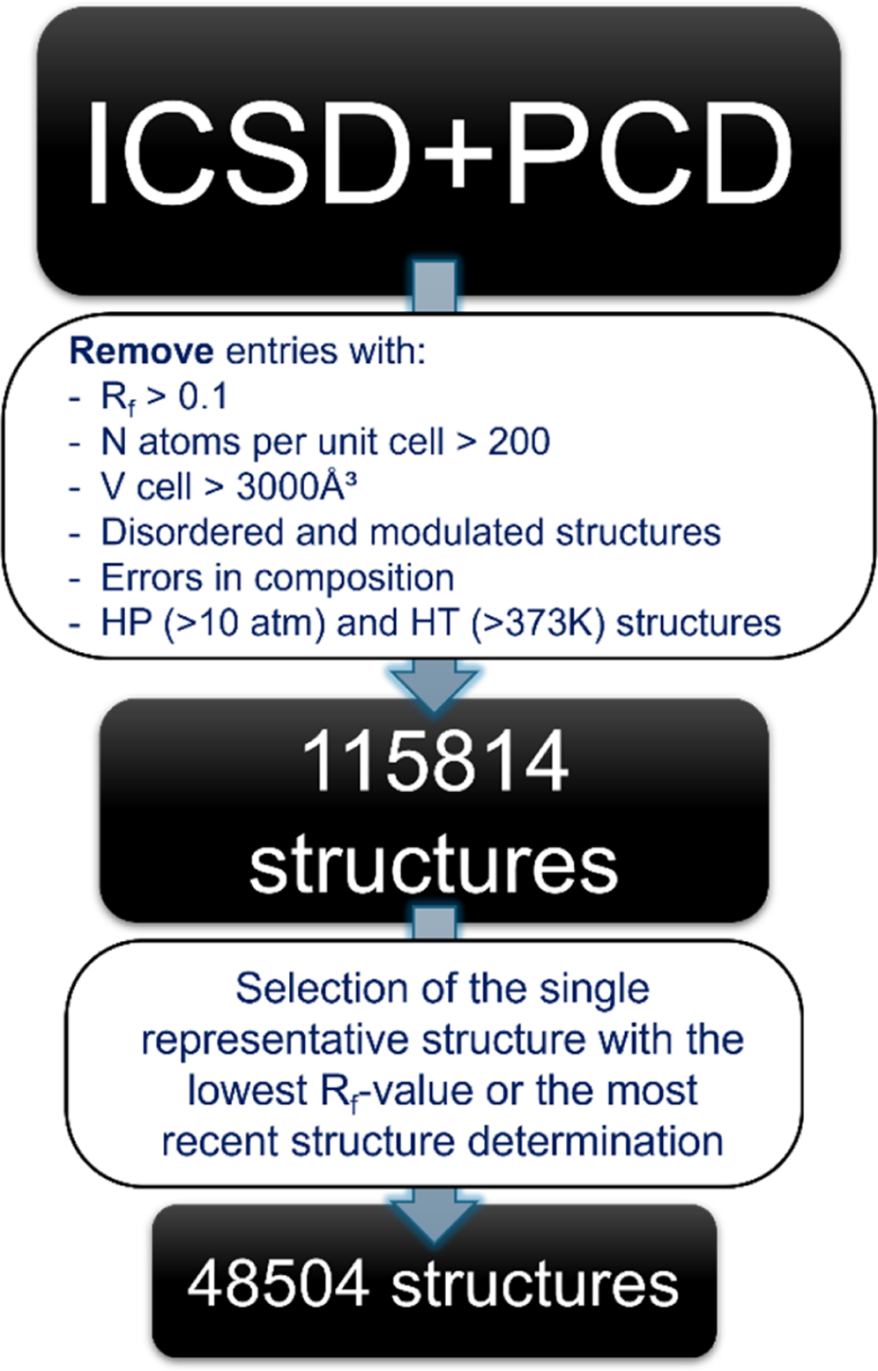
A flowchart diagram of the process for filtering the crystal structures from the ICSD and PCD databases.

Upon completion of the duplicate removal process, the final data set comprised a curated collection of representative crystal structures, with each structure representing a unique and distinct crystal structure polymorph. These structures subsequently underwent further refinement and validation processes. Notably, special emphasis was placed on structures containing CH, NH, OH, FH, and SH bonds, since the lengths of these bonds are usually underestimated in X-ray diffraction studies. A dedicated renormalization procedure was performed to adjust the hydrogen atom coordinates within these structures, aligning them with the idealized El–H bond lengths. For instance, standard bond lengths of 1.09 Å for C–H, 1.01 Å for N–H, 0.99 Å for O–H, 0.95 Å for F–H, and 1.33 Å for S–H were utilized. Bond length resetting takes place if it is smaller than the idealized value minus 0.05 Å. When such a deviation is detected, the H atom coordinates are adjusted to match the idealized bond lengths typically obtained from neutron diffraction studies.

### Ab Initio Calculations

We use the Quantum ESPRESSO^40,41^ package with the SSSP PBE efficiency pseudopotentials library (version 1.3),^42^ a library of tested pseudopotentials from different sources.^43–47^ For each material, the wave function and charge-density cutoffs are chosen as the highest suggested by the SSSP library for all of the elements in the compound. For bulk materials, we use two properly vdW-corrected functionals, namely, the vdW-DF2 functional^48^ with c09 exchange.^49^ When optimizing and computing the electronic properties of isolated 2D units, a vacuum space of 20 Å is used along the orthogonal direction and a variable cell relaxation involving only the in-plane coordinates is performed under open-boundary conditions^50^ and using the PBE^51^ functional. Sampling of the Brillouin zone is performed using a Γ-centered Monkhorst–Pack grid,^52^ with the smallest number of *k*-points in each direction of the reciprocal lattice guaranteeing a spacing of at least 0.2 Å^–1^ using a Marzari–Vanderbilt–DeVita–Payne cold smearing^53^ of 0.02 Ry. Band structures are computed along high-symmetry paths with a *k*-point density of 0.01 Å^–1^. All of the structures, even the ones with atoms that might allow a magnetically ordered ground state, are treated as nonmagnetic in a spin-unpolarized approximation. All the ab initio calculations have been run through the AiiDA infrastructure to ensure the full reproducibility of the study.^54,55^

## Supplementary Material







## Data Availability

The proposed algorithm, implemented in the CrystalSubstructureSearcher code, is freely available at the following GitHub repository: https://github.com/trioxane/CrystalSubstructureSearcher. All additional data supporting the findings of this work are provided in the Supporting Information and are also available through the MaterialsCloud web platform (10.24435/materialscloud:f1-2k).

## Notes

Plots with additional data on the ML estimation of *R*_0_ BV parameters; RLM fit lines for *E*_bind_/*A* vs BVS/A dependencies, comparison of the interlayer BVS/A descriptor calculated for both experimental and DFT-optimized crystal structures used in the creation of the MC2D database; tables with RLM regression coefficients and deduced threshold BVS/A for each contact type; and flowchart diagram of the CrystalSubstructureSearcher code (PDF)
Archive containing CIF files in the following directories: input_MC2D_filenames_(DFT_relaxed)—DFT-relaxed crystal structures used for the creation of the MC2D database by means of the distance-based algorithm; CSS_identified_2p_substructure—2-periodic substructures identified using the CrystalSubstructureSearcher code; layers are standardized by reorienting perpendicular to the *z*-axis; input CIF files for the calculation were taken from the input_MC2D_filenames_(DFT_relaxed) directory; CSS_identified_prospective_2p_substructure—2-periodic substructures identified using the CrystalSubstructureSearcher code, obtained from the analysis of the curated ICSD subset; test_set_162—DFT-relaxed crystal structures for which 2-periodic substructures were identified by means of the distance-based algorithm; and dft_analysis_new_robust_layers—folder containing results of DFT modeling of newly identified 2D materials (7Z) (ZIP)
Electronic spreadsheets file containing data on the following sheets: identified_layers, no_layers_identified, different_layers_identified—results of the CrystalSubstructureSearcher analysis of the data set of 1713 DFT-relaxed crystal structures used for the creation of the MC2D database; Ebinding_calc_vs_estimate—comparison of the interlayer binding energy estimates obtained using the RLM model with DFT-computed binding energy; new_prospective_exfoliable—list of the potentially exfoliable crystal structures containing layered substructures with composition previously not found in the databases of 2D materials; non_vdW_2D—results of the CrystalSubstructureSearcher analysis of the crystal structures exfoliation of which results in nonvan der Waals 2D materials; and ML_BV_parameters—the CatBoost regressor estimates of the correction factor Δ_ij_ and corresponding calculated *R*_0_ BV parameter for the set of all 4851 atomic pairs in the set of 98 elements (XLSX)
