# Supplementary material for: Search for Low-Periodic Substructures in Crystalline Solids: A Novel Approach
Source: ACS Appl Mater Interfaces. 2026 May 5;18(19):27997–8008. doi: 10.1021/acsami.6c03558 (PMC13195581; doi:10.1021/acsami.6c03558)
Supplement: Supplementary file 1 [file am6c03558_si_001.pdf]

Supporting Information

Search for Low-Periodic Substructures in Crystalline Solids: A Novel Approach

Pavel N. Zolotarev,[a] Davide M. Proserpio,[b] Davide Campi[a]**

[a] Dipartimento di Scienza dei Materiali, Università degli Studi di Milano-Bicocca, Via R. Cozzi 55, 20125 Milano, Italy
[b] Dipartimento di Chimica, Università degli Studi di Milano, Via Golgi 19, 20133 Milano, Italy

*E-mail: pavel.zolotarev@unimib.it

*E-mail: davide.campi@unimib.it

Table of Contents

Figures S1-S9 .... **2-8**

Tables S1-S6 .... **9-12**

Scheme S1 .... **13**

Sensitivity analysis of the filtering procedure **14-15**

Interlayer interatomic contact aggregation error estimation **16**

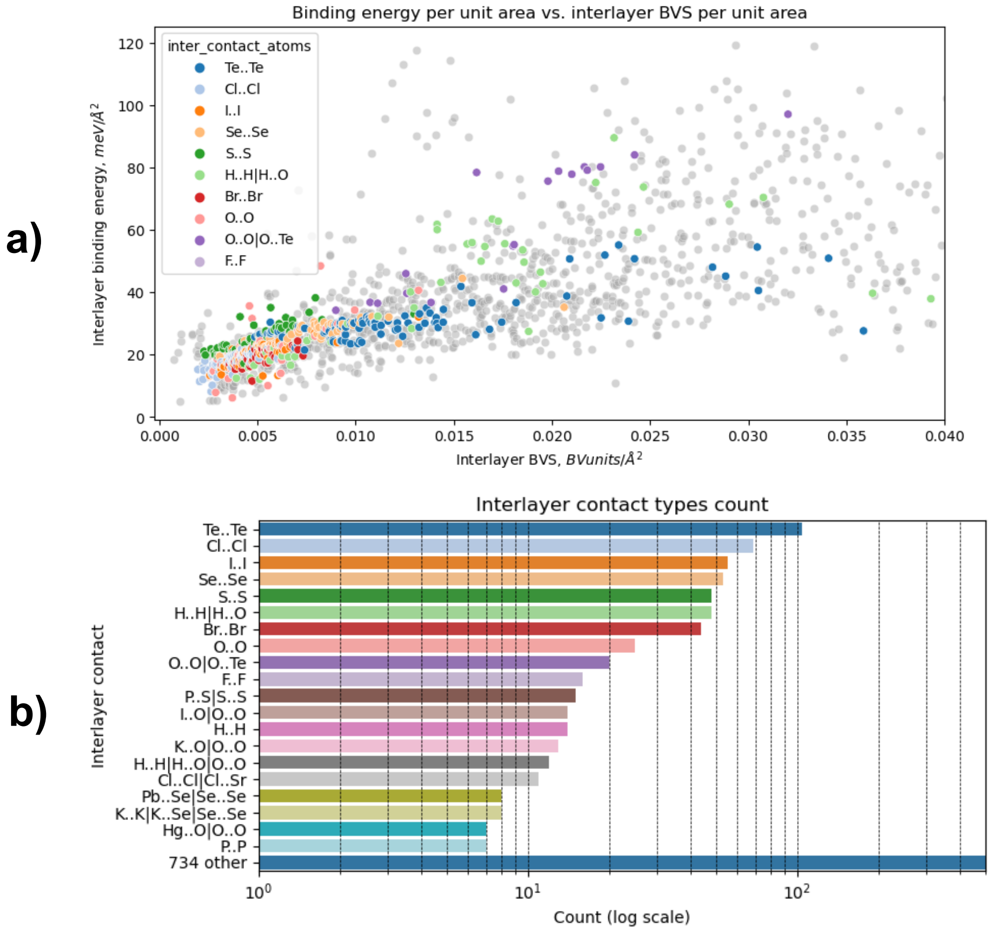

Figure S1. (a) Scatterplot of the E_{bind}/A vs BVS/A for different interfacial interatomic contacts found in the crystals from the data set. Coloured points correspond to the contacts between layers encountered in more than 15 structures. The light gray points correspond to structures with more rarely encountered interlayer contacts. (b) Countplot of the number of crystal structures with specific interlayer contact types combinations.

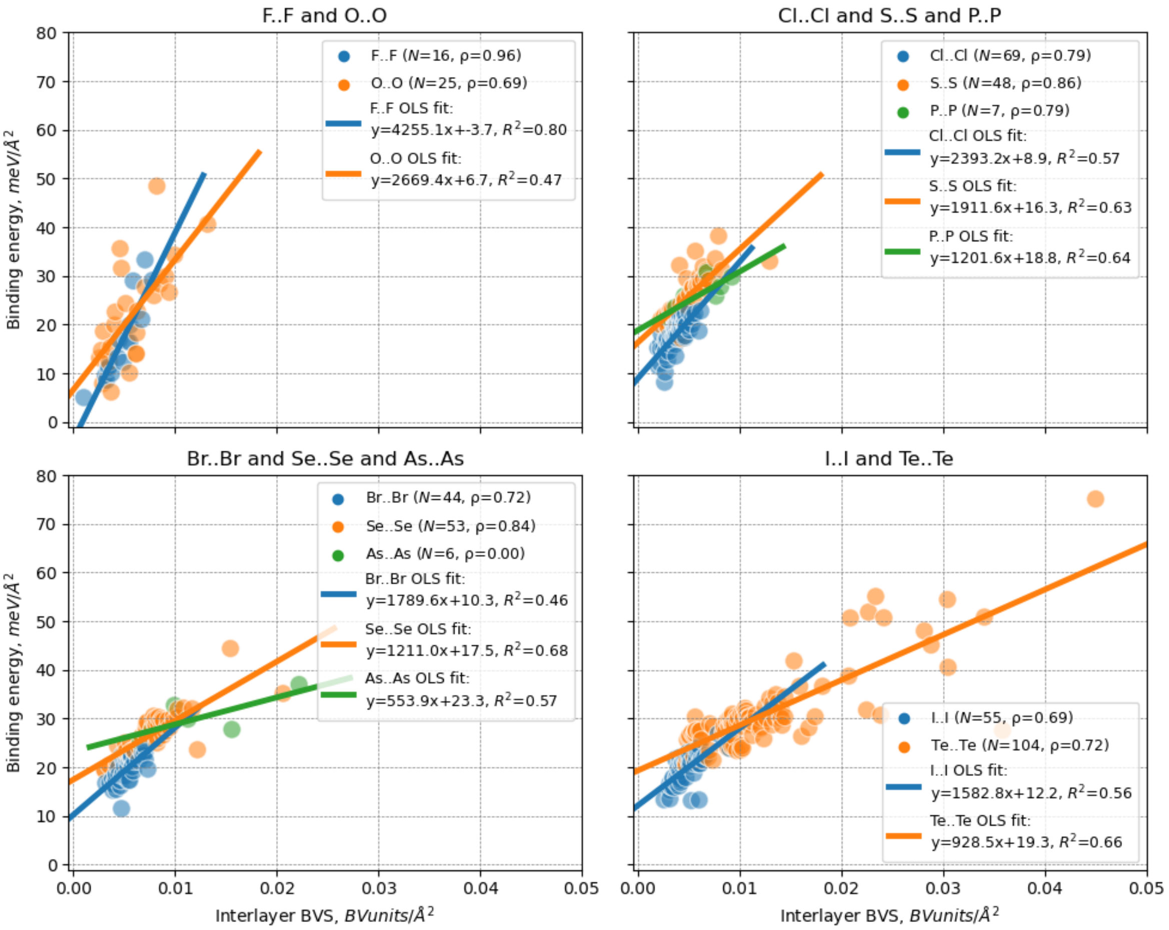

Figure S2. Ordinary Least Squares (OLS) fit lines of the binding energy per unit area as a function of the BVS per unit area for the interfacial homoatomic contacts formed by group 15/16/17 atoms from the same period. Modelling of the binding energy per unit area vs BVS per unit area relationship with OLS fitted lines allows one to reveal several regularities. For the contacts formed by atoms in the same period the intercept values increase in a row Hal..Hal < Ch..Ch < Pn..Pn. For instance, for the third period elements the line intercepts increase as 8.9 (Cl..Cl) < 16.3 (S..S) < 18.8 (P..P). At the same time, the line slope increases in the reverse order Pn..Pn < Ch..Ch < Hal..Hal, parallel to the increase in the single covalent bond strength (see Table S6), as exemplified by the third period elements 1201 (P..P) < 1911 (S..S) < 2393 (Cl..Cl). The differences in the intercepts and slopes lead to the intersection of the lines at values of BVS per unit area descriptor close to 0.01. This intersection corresponds to the inversion of the bonding strength of the Pn..Pn / Ch..Ch / Hal..Hal contacts. At low values of BVS per unit area the strength of the contacts decreases from Pn..Pn to Hal..Hal, while for the higher values of BVS per unit area strength of the contacts changes the order such that in a row Pn..Pn < Ch..Ch < Hal..Hal contacts become progressively stronger. This could be explained by the fact that lower BVS per unit area values correspond to longer and dispersion-dominated contacts, which are stronger for more polarizable atoms (see Table S6). On the contrary, higher BVS per unit area values correspond to the contacts more similar to covalent bonds, which are stronger for Hal..Hal pairs compared to Ch..Ch and Pn..Pn ones. Interestingly, especially for the period 3, 4, and 5 El..El contacts, the intersection point lies close to the 30 meV/Å^2 binding energy threshold that was proposed in [Mounet, N. et al *Nat Nanotechnol* **2018**, *13*, 246–252] as a borderline between dispersion-dominated "*easily exfoliable*" solids and those exhibiting "*relatively weak, possibly non-vdW, bonding*" between the layers. Scarcity of the structures with N..N interlayer contacts (3 structures) and the absence of structures with Sb..Sb interlayer contacts precluded us from fitting the OLS lines for them. The slope coefficients are statistically significant for all the fitted lines (p-value<0.05 for a hypothesis test whose null hypothesis is that the slope is zero) except for the As..As line.

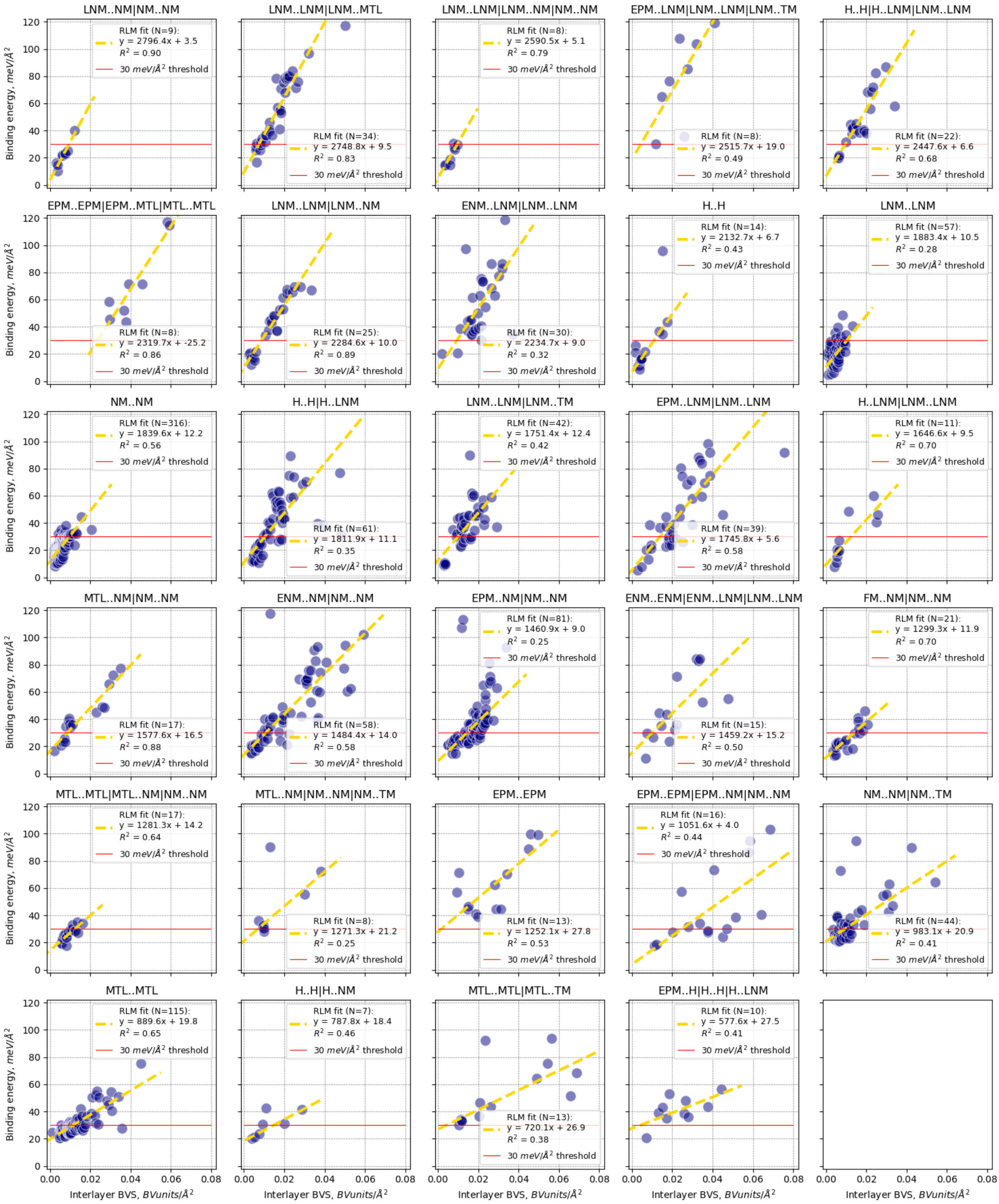

Figure S3. Scatterplots with robust linear model (RLM) fit lines for E_{bind}/A vs BVS/A dependencies for each of the 29 aggregated contact types.

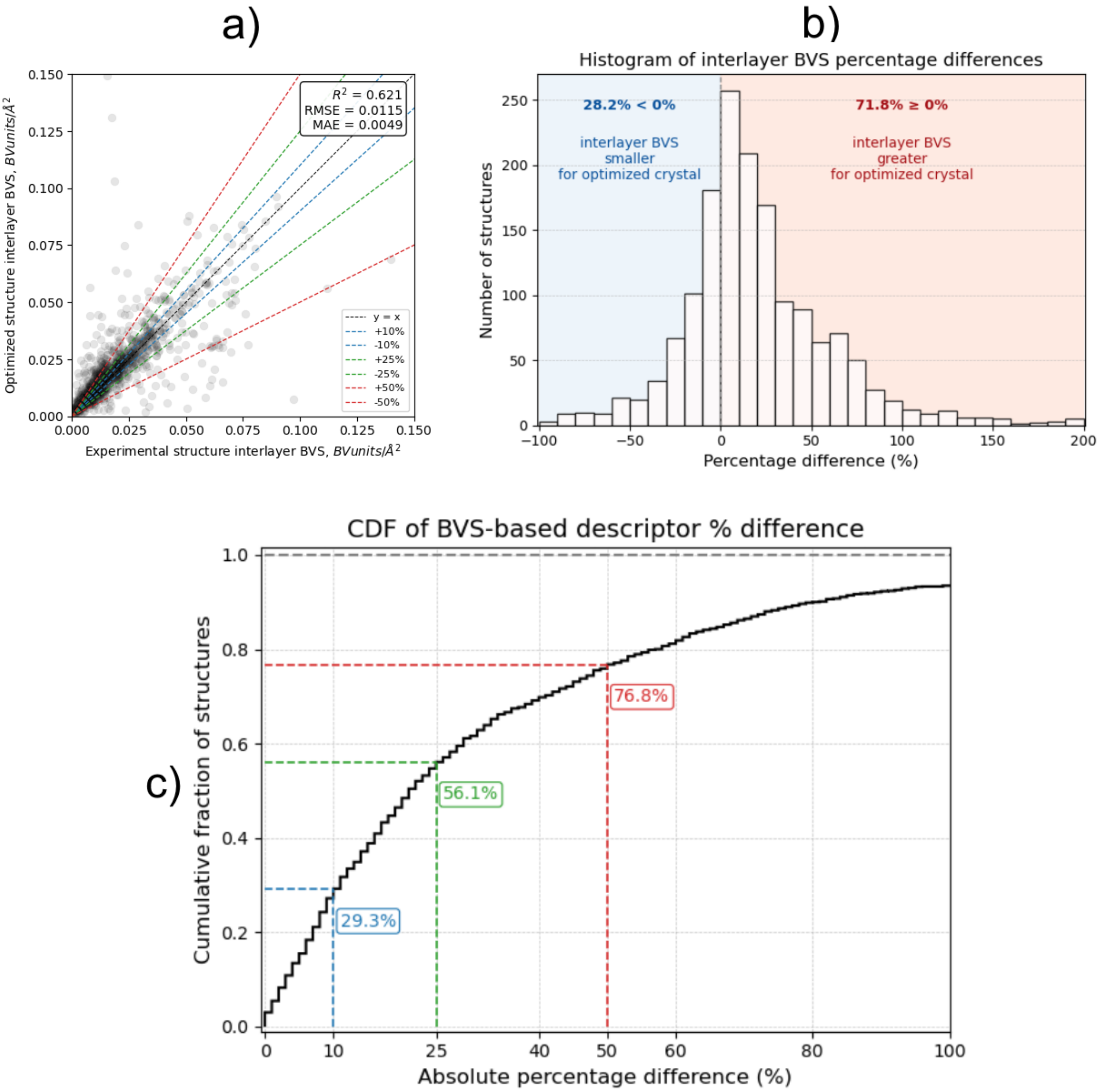

Figure S4. a) Scatterplot of the interlayer BVS/A descriptor calculated for both experimental and DFT-optimized crystal structures used in the creation of the MC2D database. b) Histogram showing the distribution of relative percentage differences between the interlayer BVS/A descriptors obtained from optimized vs experimental crystal structures. Notably, for the majority of structures (71.8%), the percentage difference is positive, indicating that DFT optimization leads to shortening of interlayer contacts. This shortening increases the corresponding bond valences and, consequently, leads to the increased BVS/A descriptor. c) Cumulative distribution function (CDF) of the absolute percentage differences between descriptors calculated from optimized and experimental structures. Threshold values of 10%, 25%, and 50% are highlighted to illustrate the proportion of structures exhibiting BVS/A descriptor differences less than or equal to the corresponding value.

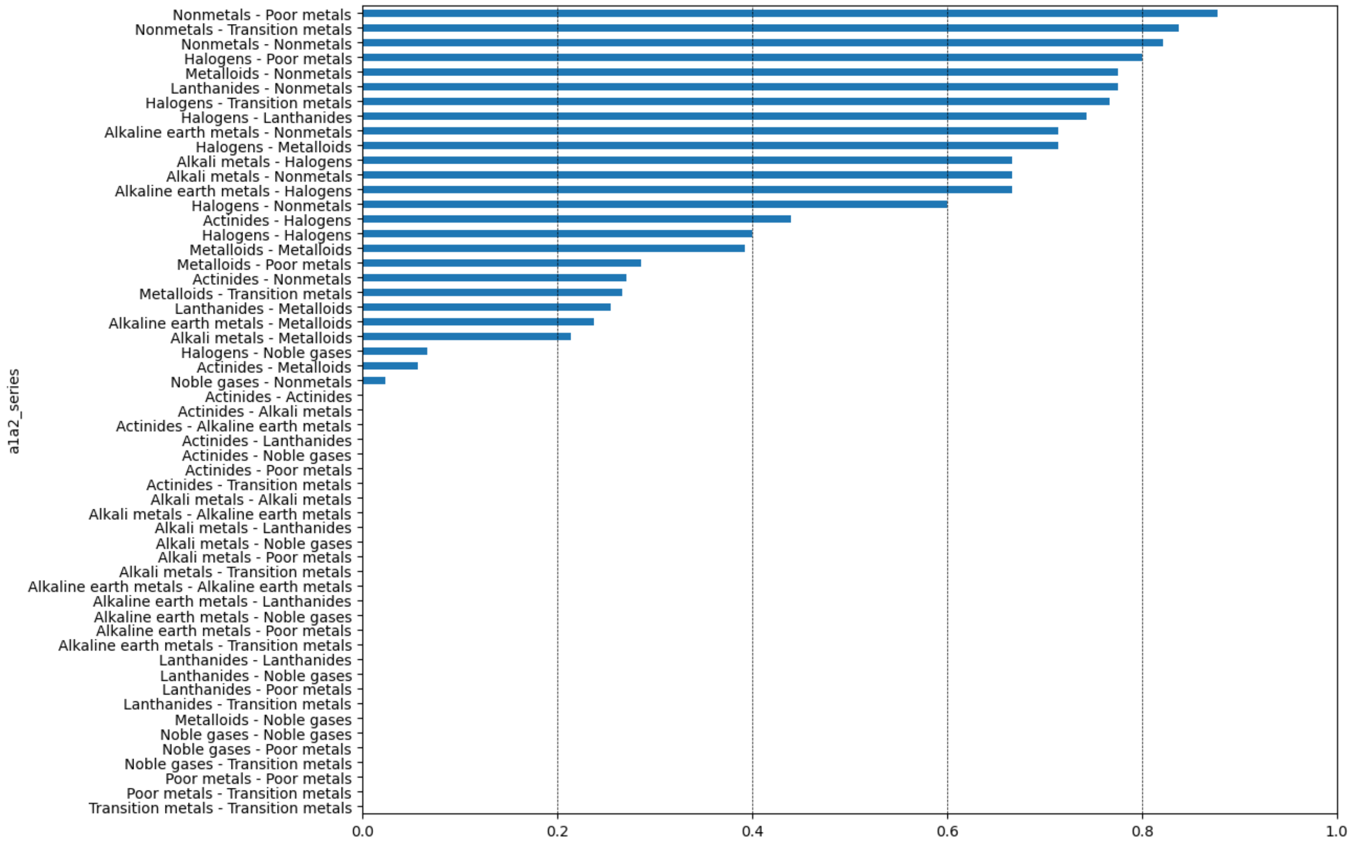

Figure S5. The barplot shows for a pair of atoms of a given type (alkali metals, transition metals, halogens, etc.) forming a contact share of the contacts between atoms for which BV parameters are available in [*https://www.iucr.org/resources/data/datasets/bond-valence-parameters*] relative to all possible combinations of atoms of a given type. For example, the R_0 parameter is available only for 6 Hal..Hal contacts (Cl..F, Br..F, I..F, Cl..Cl, Br..Cl, I..Cl) that amounts to 40% of all possible $C_5^2 + 5 = 15$ combinations of 5 halogen atoms F, Cl, Br, I, At. The atom types used are as defined in the *mendeleev* python library.

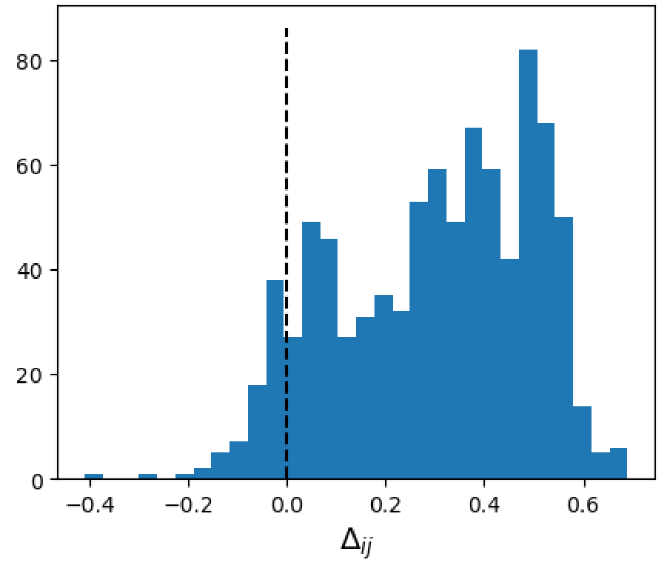

Figure S6. Distribution of the Δ_{ij} values in the dataset. Most of the values are positive meaning that R_0 parameter is usually smaller than the sum of the element covalent radii.

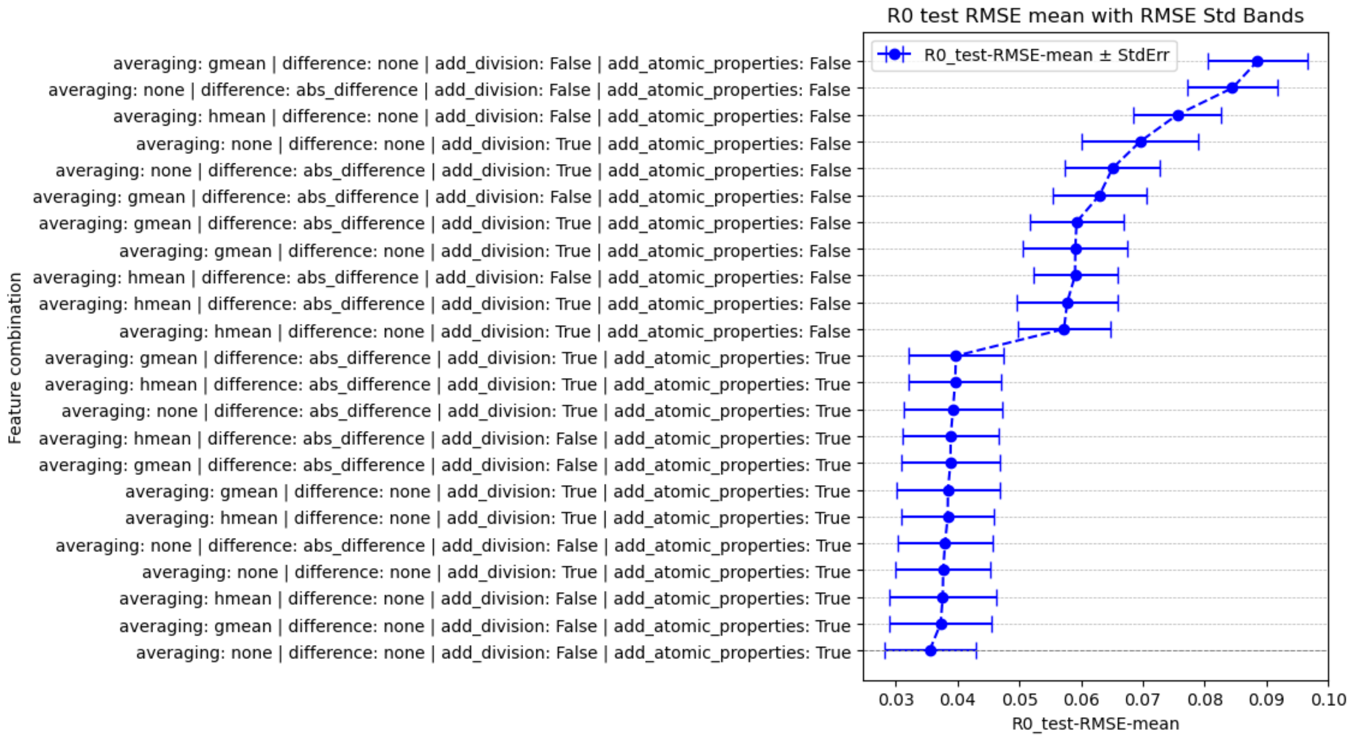

Figure S7. R_0 parameter prediction test set RMSE values with standard error bars for each feature combination tested.

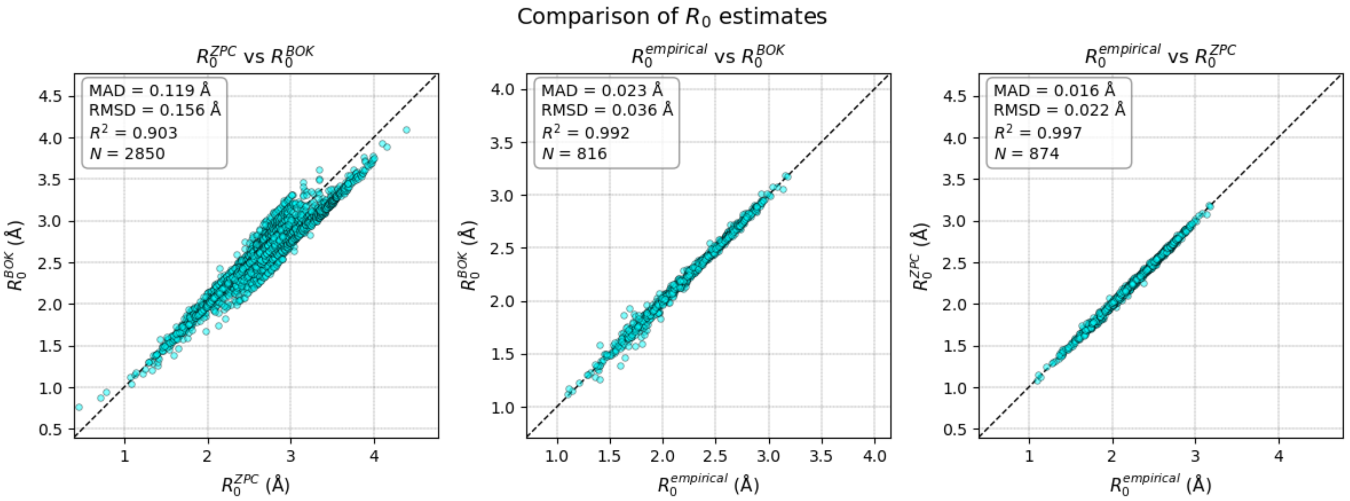

Figure S8. Comparison of the estimated R_0 (by two methods) values against the ground truth R_0 empirical values. ZPC and BOK – correspond to the sets of R_0 BV parameters from the current study and reference 28, respectively. The BOK R_0 parameters were calculated using the *r* and *c* values for elements taken from the *src/pymatgen/core/bvparam_1991.yaml* file in the *pymatgen* GitHub repository.

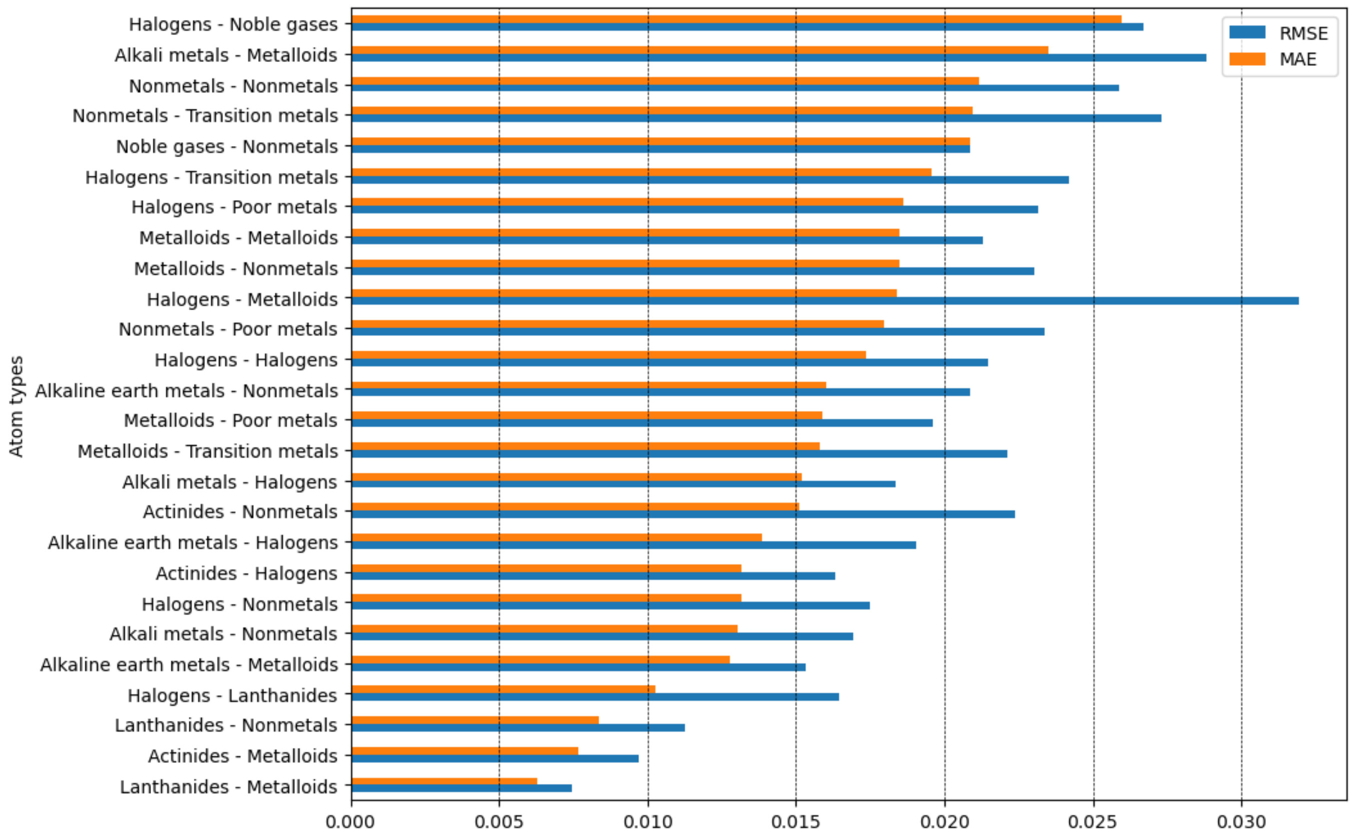

Figure S9. RMSE and MAE of the estimated R_0 values against the ground truth R_0 empirical values for separate bond groups.

Table S1. List of the substructure and interface descriptors computed by the CrystalSubstructureSearcher. Note that the BVS descriptors are calculated for the 1x1x2 supercell.

descriptor	units	description	Ca_2Sb (ICSD 154)
total_bvs	BV units	Total sum of bond valences in the cell	36.556
intra_bvs	BV units	BVS that correspond to contacts between the atoms belonging to the components of the structure graph	30.069
inter_bvs	BV units	BVS that correspond to contacts between the components of the structure graph	6.487
xbvs	fraction	Fraction of BVS in the components with target periodicity with respect to the *total_bvs*	0.823
BVS_x_periodicity	BV units	*intra_bvs* values for each of the identified low-periodic substructure stored as a dictionary	{3: 36.556, 2: 30.069, 0: 2.412}
3-p	fraction	Intrinsic 3-periodicity fraction	0.190
2-p	fraction	Intrinsic 2-periodicity fraction	0.810
1-p	fraction	Intrinsic 1-periodicity fraction	0.000
mean_inter_bv	BV units	Mean bond valence of contacts between the components of the structure graph	0.2027
inter_bvs_per_interface	BV units	BVS for contacts between the components of the structure graph per interface	1.6218
inter_bvs_per_unit_area (BVS/A)	BV units/Å^2	Calculated only for 2-periodic components by division of *inter_bvs_per_interface* over hkl_area reflecting the strength of the intercomponent binding	0.0737
inter_contact_atoms		Atom pairs forming inter component contacts	Ca..Ca|Ca..Sb
inter_contact_atoms_count		Counts of atom pairs forming inter component contacts	{'Ca..Sb': 16, 'Ca..Ca': 16}
inter_contact_arbitrary_types		Aggregated atom pairs forming inter component contacts	EPM..EPM|EPM..MTL
inter_contact_arbitrary_types_count		Counts of aggregated atom pairs forming inter component contacts	{'EPM..MTL': 16, 'EPM..EPM': 16}
estimated_charge	q_e	Estimated charge of the substructure components obtained using the electronegativity differences of the atoms forming contacts between different components of the SG	0.0

Table S2. OLS regression coefficients and deduced threshold BVS/A values for each contact type.

contact	N	slope	intercept	R^2	slope pvalue	slope stderr	threshold BVS/A
H..O|O..O	6	5829.3	-17.0	0.994	1.5E-05	230.7	0.008
F..F	16	4255.1	-3.7	0.805	2.5E-06	560.2	0.008
F..F|F..Pb	5	3694.8	-18.5	0.830	3.1E-02	965.3	0.013
P..S|S..S	15	3529.4	7.4	0.539	1.8E-03	905.9	0.006
H..H	14	2952.1	5.9	0.527	3.3E-03	807.7	0.008
O..O	25	2669.4	6.7	0.469	1.6E-04	592.7	0.009
K..O|O..O	13	2640.6	-10.6	0.768	8.6E-05	438.0	0.015
Cl..Cl	69	2393.2	8.9	0.570	6.6E-14	253.9	0.009
O..O|O..Te	20	2358.9	17.8	0.779	2.6E-07	296.0	0.005
Bi..O|O..O	5	2330.8	4.9	0.927	8.5E-03	376.4	0.011
Bi..Cl|Cl..Cl	6	2207.6	5.0	0.985	8.2E-05	134.9	0.011
S..S	48	1911.6	16.3	0.633	1.4E-11	214.6	0.007
H..H|H..O|O..O	12	1852.5	15.4	0.530	7.3E-03	551.7	0.008
Br..Br	44	1789.6	10.3	0.460	4.2E-07	299.1	0.011
I..O|O..O	14	1777.0	20.8	0.814	1.0E-05	245.0	0.005
I..I	55	1582.8	12.2	0.557	6.2E-11	193.9	0.011
P..Se|Se..Se	5	1448.9	16.5	0.884	1.7E-02	303.3	0.009
K..K|K..Se|Se..Se	8	1338.5	-2.7	0.507	4.8E-02	538.8	0.024
Cl..Cl|Cl..O	5	1217.9	14.2	0.943	5.8E-03	172.3	0.013
Se..Se	53	1211.0	17.5	0.678	3.6E-14	116.7	0.010
P..P	7	1201.6	18.8	0.642	3.0E-02	401.7	0.009
H..H|H..O	48	1169.0	21.9	0.377	3.5E-06	221.6	0.007
Te..Te	104	928.5	19.3	0.660	1.3E-25	66.0	0.011
Br..Br|Br..Sr	6	819.3	15.0	0.787	1.8E-02	213.0	0.018
Ca..Cl|Cl..Cl	7	748.3	19.2	0.953	1.7E-04	74.6	0.014
As..Li|Li..Li	5	-1129.2	116.5	0.967	2.6E-03	121.1	0.077

Table S3. Arbitrarily defined coarse groups of elements. ENM – electronegative metals; EPM – electropositive metals; FM – *f*-block metals; H – hydrogen (treated as a separate group due to its unique properties); LNM – light non-metals (non-metals from the second period); MTL – metalloids; NG – noble gases; NM – the rest of the non-metals; TM – *d*-block metals.

Group	Elements
ENM	Al, Be, Bi, Fl, Ga, In, Lv, Mc, Nh, Pb, Sn, Tl
EPM	Ba, Ca, Cs, Fr, K, Li, Mg, Na, Ra, Rb, Sr
FM	Ac, Am, Bk, Ce, Cf, Cm, Dy, Er, Es, Eu, Fm, Gd, Ho, La, Lr, Lu, Md, Nd, No, Np, Pa, Pm, Pr, Pu, Sm, Tb, Th, Tm, U, Yb
H	H
LNM	C, F, N, O
MTL	As, B, Ge, Po, Sb, Si, Te
NG	Ar, He, Kr, Ne, Og, Rn, Xe
NM	At, Br, Cl, I, P, S, Se, Ts
TM	Ag, Au, Bh, Cd, Cn, Co, Cr, Cu, Db, Ds, Fe, Hf, Hg, Hs, Ir, Mn, Mo, Mt, Nb, Ni, Os, Pd, Pt, Re, Rf, Rg, Rh, Ru, Sc, Sg, Ta, Tc, Ti, V, W, Y, Zn, Zr

Table S4. RLM regression coefficients and deduced threshold BVS/A values for each aggregated contact type. Note that the threshold values were increased by 20% so that more structures on the verge of the arbitrarily selected borderline of 30 meV/Å^2 could be accepted.

contact group	N	slope	intercept	R^2	slope pvalue	slope stderr	threshold BVS/A
EPM..EPM	13	1252.1	27.8	0.529	4.9E-04	358.9	0.002
EPM..H|H..H|H..LNM	10	577.6	27.5	0.411	3.7E-02	276.2	0.005
MTL..MTL|MTL..TM	13	720.1	26.9	0.380	7.5E-08	133.9	0.005
EPM..LNM|LNM..LNM|LNM..TM	8	2515.7	19.0	0.494	4.4E-03	883.1	0.005
MTL..NM|NM..NM|NM..TM	8	1271.3	21.2	0.250	2.6E-10	201.2	0.008
LNM..LNM|LNM..MTL	34	2748.8	9.5	0.834	4.0E-55	175.8	0.009
MTL..NM|NM..NM	17	1577.6	16.5	0.884	1.6E-23	157.8	0.010
LNM..LNM|LNM..NM	25	2284.6	10.0	0.888	5.1E-56	144.9	0.010
NM..NM|NM..TM	44	983.1	20.9	0.408	3.6E-19	109.9	0.011
ENM..LNM|LNM..LNM	30	2234.7	9.0	0.319	3.6E-10	356.4	0.011
LNM..NM|NM..NM	9	2796.4	3.5	0.904	5.8E-11	426.9	0.011
H..H|H..LNM|LNM..LNM	22	2447.6	6.6	0.681	6.2E-15	313.8	0.011
LNM..LNM|LNM..NM|NM..NM	8	2590.5	5.1	0.787	4.5E-06	565.0	0.012
NM..NM	316	1839.6	12.2	0.559	3.0E-127	76.7	0.012
LNM..LNM|LNM..TM	42	1751.4	12.4	0.418	4.2E-11	265.5	0.012
ENM..ENM|ENM..LNM|LNM..LNM	15	1459.2	15.2	0.500	4.7E-04	417.2	0.012
LNM..LNM	57	1883.4	10.5	0.276	1.3E-05	431.1	0.012
H..H|H..LNM	61	1811.9	11.1	0.352	1.1E-28	163.1	0.013
ENM..NM|NM..NM	58	1484.4	14.0	0.577	9.0E-47	103.4	0.013
H..H	14	2132.7	6.7	0.433	1.1E-22	217.5	0.013
MTL..MTL	115	889.6	19.8	0.655	7.3E-88	44.8	0.014
MTL..MTL|MTL..NM|NM..NM	17	1281.3	14.2	0.644	5.4E-07	255.7	0.015
H..LNM|LNM..LNM	11	1646.6	9.5	0.705	1.7E-06	344.4	0.015
FM..NM|NM..NM	21	1299.3	11.9	0.705	1.1E-10	201.5	0.017
EPM..LNM|LNM..LNM	39	1745.8	5.6	0.582	7.5E-18	202.8	0.017
EPM..NM|NM..NM	81	1460.9	9.0	0.248	1.6E-26	137.1	0.017
H..H|H..NM	7	787.8	18.4	0.455	2.3E-04	213.7	0.018
EPM..EPM|EPM..MTL|MTL..MTL	8	2319.7	-25.2	0.855	4.2E-06	504.4	0.029
EPM..EPM|EPM..NM|NM..NM	16	1051.6	4.0	0.438	9.7E-04	318.7	0.030

Table S5. Hyperparameters used for the CatBoost regressor training when searching for the best feature representation of the bonds.

Parameter	Value
loss_function	RMSE
random_seed	23
iterations	500
learning_rate	0.05
depth	5
min_data_in_leaf	7
nan_mode	Min
subsample	0.8

Table S6. Atomic polarizabilities in Å^3 [Schwerdtfeger, P., & Nagle, J. K. (2019). *Molecular Physics*, *117*(9-12), 1200-1225] and single El-El covalent bond dissociation energies in kJ/mol [https://www.wiredchemist.com/chemistry/data/bond_energies_lengths.html] for a set of Pn..Pn, Ch..Ch and Hal..Hal homoatomic contacts.

**Interatomic Contacts**	**Atomic Polarizability (Å^3)**	**Single El-El Bond Energy (kJ/mol)**
F..F	3.75	155
O..O	5.3	142
Cl..Cl	14.6	240
S..S	19.4	226
P..P	25	201
Br..Br	21	190
Se..Se	29	172
As..As	30	147
I..I	33	149
Te..Te	38	126

Scheme S1. Overview of the algorithm for the search of the low-periodic substructures represented as a flowchart diagram.

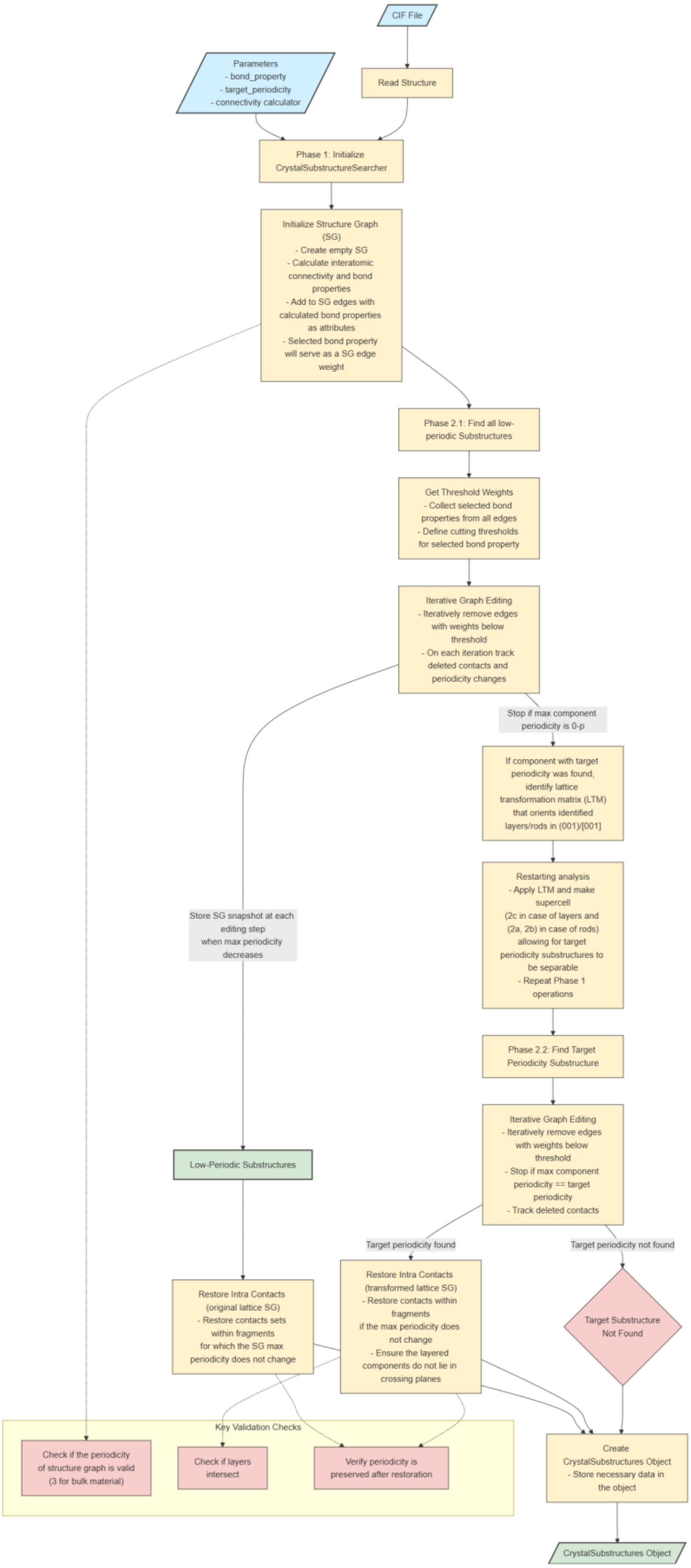

Sensitivity analysis of the filtering procedure

The thresholds employed in this study were determined through systematic analysis of structures that successfully met the criteria for inclusion in the MC2D database. Specifically, the BVS per unit area values for the 29 aggregated contact types enabled exfoliability assessment for 8343 structures out of 27350 candidates, of which 2135 exhibited BVS\A lower than the threshold corresponding to 30 meV/Å^2 plus a safety margin to account for the possible errors in our estimation. The original choice of 30 meV/Å^2 is somehow empirical based on the clustering behavior of binding energies and the values of the computed binding energies corresponding to materials experimentally isolated through mechanical exfoliation (like graphene, phosophrene, MoS_2, InSe, BN, $PtSe_2$) as reported in Fig.2 of [ref1].

Subsequently, structures with charged substructures were filtered out, yielding a final set of 1989 potentially easily exfoliable crystal structures with neutral substructures (estimated charge $< 0.25|q_e|$). This charge threshold was selected based on the maximal absolute charges observed in layers of the easily exfoliable subset of the MC2D database. Below we show the plot with cumulative count of structures with estimated charge less than threshold value.

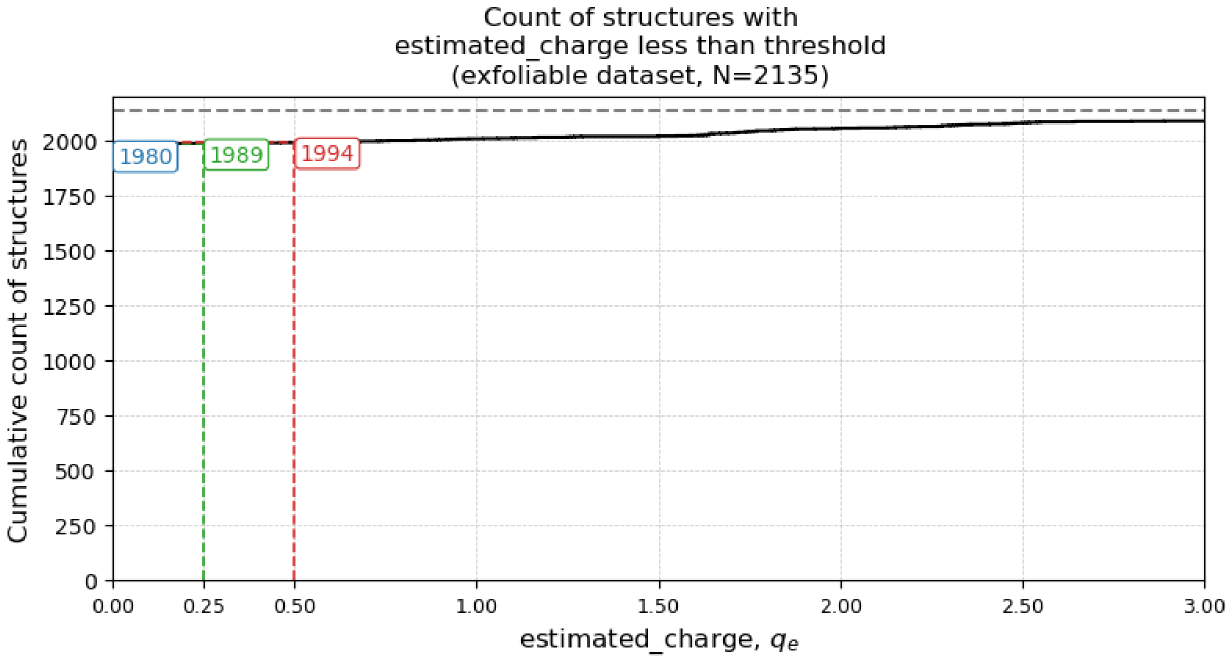

Finally, the definition of "robust" layers is then based on a BV based descriptor that we named *max_intracomponent_bond_strength*. Below are shown cumulative counts of structures with values of *max_intracomponent_bond_strength* descriptor less than a given threshold. The first plot refers to the 1633 crystal structures used for the creation of the MC2D database. We can see that 99% of the crystal structures in which layers were identified by the CSS code have *max_intracomponent_bond_strength* descriptor larger than 0.1 BV units and how there is a distinct change in the count slope around this value.

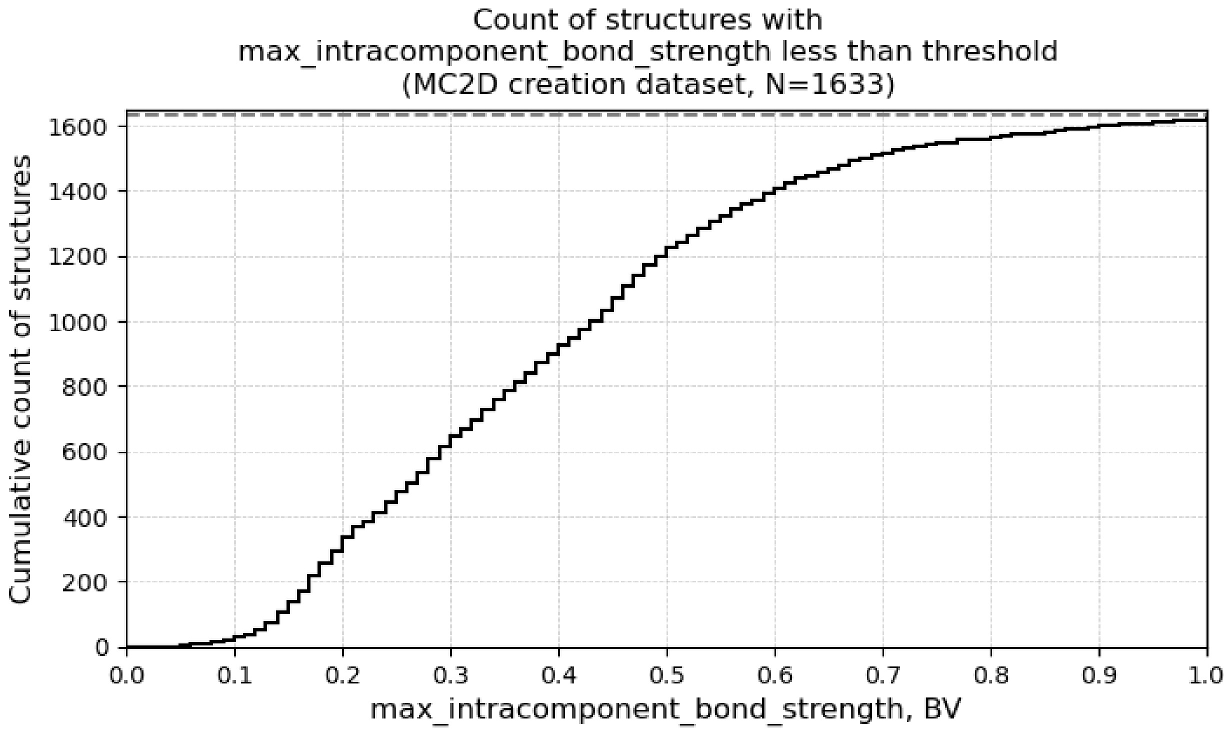

Therefore, we selected this threshold for the subsequent filtering of the potentially exfoliable set of 963 crystal structures to obtain a shortlist of 712 crystal structures with layered substructures of sufficient robustness. Below we demonstrate how many crystal structures would have been filtered out depending on the *max_intracomponent_bond_strength* thresholds selection.

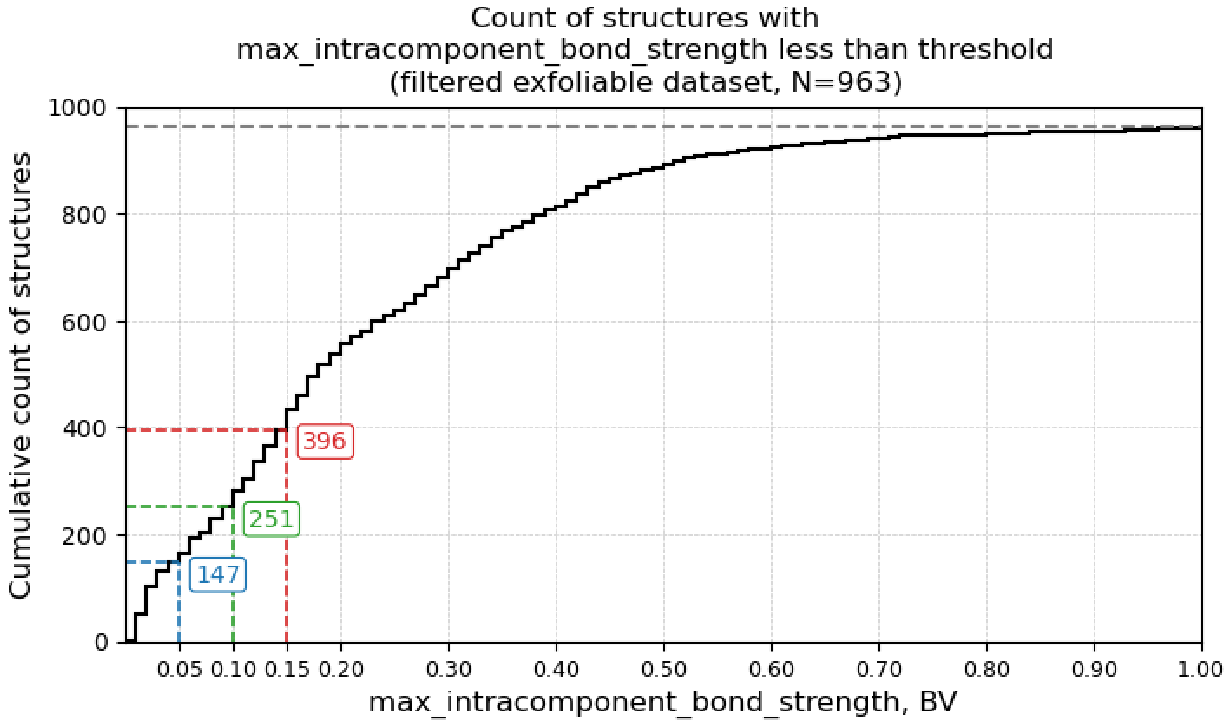

Interlayer interatomic contact aggregation error estimation

The grouping was applied following chemical resemblances to minimize the possibility of obscuring bonding environments with distinctly different chemical behavior. However, the grouping strategy might smear out some chemical differences within the groups. To estimate the potential errors introduced by the grouping we compared the binding energies computed for the non aggregated contact types in the NM..NM group (that is for separate contact types Cl..Cl, S..S, I..I, etc.) at the NM..NM group threshold BVS/A value corresponding to 30 meV/A^2. The selected NM..NM aggregated group is the only one that comprises sufficient number of separate contact types to provide a statistically meaningful ensemble.

	contact	Eb	ΔEb
0	P..S|S..S	41.555401	11.554793
1	S..S	34.812877	4.812269
2	Cl..Cl	32.103495	2.102887
3	P..P	30.460240	0.459633
4	P..Se|Se..Se	30.531152	0.530544
5	Se..Se	29.229236	-0.771372
6	Br..Br	27.604934	-2.395674
7	I..I	27.497802	-2.502806
8	Te..Te	28.328477	-1.672131
9	NM..NM	30.000608	0.000000

The mean error in the estimated binding energies obtained from the linear relationships in the two cases is around 1.4 meV/A^2 (SD=4.5 meV/A^2) as shown in the table above. Generally, the grouping strategy employed is sufficiently accurate for the large-scale screening purposes.
